# Seasonal modulation of salinity stress response in leaf micro-morphological and biochemical insights of the mangrove *Avicennia* sp. (Acanthaceae) in Digha Mohona, West Bengal

**DOI:** 10.3389/fpls.2026.1769745

**Published:** 2026-03-26

**Authors:** Arpita Maity, Amal Kumar Mondal

**Affiliations:** 1 Centre for Life Science, Vidyasagar University, Midnapore, India; 2 Plant Taxonomy, Biosystematics and Molecular Taxonomy Laboratory, Department of Botany and Forestry, Vidyasagar University, Midnapore, West Bengal, India

**Keywords:** compatible osmolytes, glycine betaine, proline, salt gland index, seasonal changes, sugar alcohol

## Abstract

Seasonal variations (pre-monsoon and monsoon) cause minor shifts in the mangrove microenvironment that includes average temperature, pH, salinity, total dissolved solids, and electrical conductivity in both surface water and soil. These fluctuations lead to subtle alterations in mangrove micro-morphology (salt gland index) and influence the accumulation of compatible osmolytes (CO) such as proline, glycine betaine (GB), and sugar alcohols (mannitol and sorbitol). Seasonal changes also affect plant pigments, soluble sugars, and the accumulation of secondary metabolites in plant tissues. In this study, we examined how mangrove species adapt to their microenvironment by assessing along with salt gland index, CO, plant pigment, and secondary metabolite (total phenol content (TPC), total flavonoids content (TFC), total polyphenol content (TPPC)) accumulation in plant tissues. The biochemical composition of CO, along with seasonal accumulation pattern, was also in a species-specific manner. CO levels were highest during the pre-monsoon season and lowest during the monsoon season. *A. rumphiana* showed the highest proline concentration in the pre-monsoon season, while GB accumulation was highest in *A. rumphiana* and *A. alba*. Similarly, secondary metabolite accumulation pattern, along with their seasonal variation, exhibited a species-specific manner and plant developmental phase. The highest level of TPC was found in *A. rumphiana* during pre-monsoon, whereas *A. marina* and *A. alba* displayed the highest TPC in post-monsoon season. A significant decrease was noted in the chlorophyll content in the pre-monsoon season, while soluble carbohydrate accumulation was more pronounced during the monsoon and post-monsoon seasons. These patterns show how different species have adapted to shifting environmental circumstances.

## 1 Introduction

Plants can perceive and respond to stressful situations; few species thrive in environments where high levels of abiotic stress are present (Rodriguez et al., 2008). Abiotic stress affects large areas of farmed and irrigated land, making it a major problem for global agriculture (Shrivastava and Kumar, 2015). Most studies concerning salt tolerance involve plants that have been grown in a natural saline ecosystem, but the studies involving plants that have been grown under natural saline ecosystems are more significant in understanding salt stress tolerance (Lokhande and Suprasanna, 2012; Chowdhury et al., 2023). Among the various abiotic stresses, salinity alone affects approximately 930 million hectares, about 7% of the world’s land surface (Munns and Tester, 2008). There are a number of lands affected by hyper-ionic and hyperosmotic stress due to excessive salt accumulation in coastal and estuarine regions, generally resulting from the accumulation of salt over a long period of time (Munns, 2002).

Mangroves are unique coastal ecosystems that thrive in intertidal tropical and subtropical regions by resilience to harsh environmental conditions such as high salinity, low oxygen, and temperature fluctuations (Tomlinson, 1994). This adaptation involves the specialized physiological and morphological mechanism for the exclusion of salt through salt glands on the leaves that mitigate the adverse effects of high salt concentrations on growth, photosynthesis, and water relations (Sudhir et al., 2022). These stress-driven adaptations are also associated with the production of compatible osmolytes and diverse secondary metabolites (phenols, flavonoids, alkaloids, and terpenoids) that contribute to environmental resilience and ethnopharmacological importance (Popp, 1995; Dahibhate et al., 2018; Bibi et al., 2019; Mitra et al., 2023).

Cellular compartments detect abiotic stress triggering molecular responses through regulatory proteins and receptors (Zhang et al., 2019). These signals initiate downstream gene expression, leading to compatible osmolytes and protective protein synthesis (Melo et al., 2022). Integration of environmental signals with endogenous developmental signals influences compatible osmolyte accumulation that promote plant resistance to environmental stresses. Plants can develop broader defenses against abiotic stress, including outer cuticle layer, compatible osmolytes, internal reactive species scavengers, and molecular chaperones (He et al., 2018).

Plants exhibit salinity and drought stress tolerance through integrated physiological and biochemical responses, especially the accumulation of compatible osmolytes like proline, glycine betaine, amino acids, and other solutes that maintain cellular turgor and osmotic homeostasis (Lei et al., 2006; Rosa et al., 2020; Yadav et al., 2020). Several studies have highlighted the physiological and biochemical mechanisms, along with morphological and anatomical features, that contribute to the notable tolerance of plants to abiotic stresses such as salinity, oxygen, drought, and extreme temperatures (Naz et al., 2014; Walker and Lutts, 2014). When exposed to salt, plants use a variety of defense mechanisms, such as the buildup of secondary metabolites, reactive oxygen species, and compatible solutes. Osmotic adjustment is aided by the cytosolic accumulation of solutes such as proline and glycine betaine (Xing and Rajashekar, 2001). Osmoregulation, the process of maintaining cellular turgor pressure under osmotic stress, is crucial for plant survival under drought conditions (Öztürk et al., 2020; Mehta and Vyas, 2023b). Osmotic adjustment by these non-toxic compounds protect cellular structures and enzymes from damage caused by dehydration (Chen and Murata, 2000; Rampino et al., 2006; Oliveira et al., 2012; Sewelam et al., 2016; He et al., 2018).

The presence of ROS in the environment due to abiotic stresses such as salt is harmful to cells because they cause oxidative danger for lipids, membrane proteins, and nucleic acids (Smirnoff, 1993; Gomez et al., 1999; Hernández et al., 2001). Certain levels of ROS are also involved in antioxidative protection, even though they can harm proteins, nucleic acids, and membrane lipids. While anthocyanins, which are derived from flavonoids, accumulate as a defense mechanism under stress, flavonoids function as antioxidant agents by scavenging ROS (Ashraf and Foolad, 2007; Grigore et al., 2011; Li et al., 2010). Phenolics are essential for preserving redox balance and safeguarding biological systems. The equilibrium between reactive oxygen species and phytochemicals such as flavonoids and polyphenols affects how well plants adapt to salt stress. The plant kingdom contains phenolic compounds, particularly flavonoids, which have a variety of molecular and biochemical functions. They have antioxidant qualities, function as signaling molecules, and support plant defense. Plants can withstand salt stress because phenols and flavonoids play a major role in scavenging free radicals (Apel and Hirt, 2004). Moreover, carbohydrates support the preservation of protein structure under stress, carbon storage, and radical scavenging. Chlorophylls, carotenoids, and photosynthetic efficiency can be affected by salinity (Foyer, 2018). By scavenging ROS, flavonoids function as antioxidants, whereas anthocyanins, which are derived from flavonoids, accumulate as a protective mechanism under stress (Huang et al., 2019).

Therefore, this study focused on the leaf micro-morphology and accumulation of compatible osmolyte changes to seasonal variation. In addition, understanding how *Avicennia* sp. adjusts its leaf morphology and osmolyte production in response to seasonal variations can provide insights into the broader adaptive strategies of mangroves. Considering the moderate fluctuations in salinity levels at Digha Mohona, it was hypothesized that *Avicennia* sp. has the ability to regulate salt stress tolerance in a season-specific manner through the integrated response of leaf micro-morphological and biochemical processes. Specifically, higher salinity conditions during the dry season (pre-monsoon and post-monsoon) are expected to enhance structural adjustments (e.g., stomata and salt gland density) along with increased accumulation of osmolytes and stress-related metabolites, whereas the comparatively lower salinity in the wet season (monsoon) may reduce these stress tolerance mechanisms. Therefore, seasonal changes are hypothesized to play a major role as the key environmental regulator that integrates micro-morphological modifications with physiological and biochemical stress tolerance mechanisms in *Avicennia* spp.

## 2 Materials and methods

### 2.1 Survey area data collection

**Figure 1:**
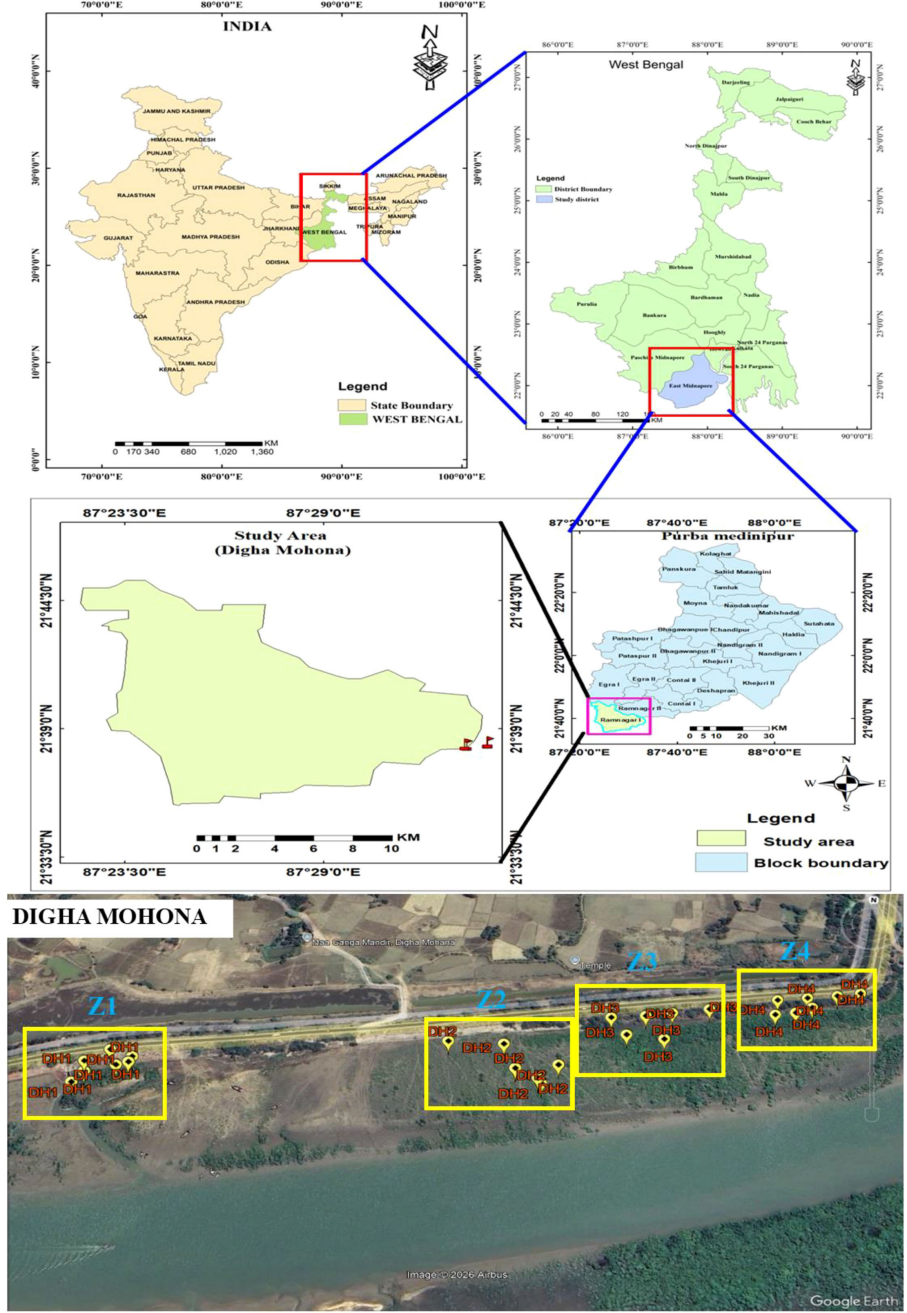
Geographical location of Digha Mohona coastal vegetation and sampling sites which surround Champa River at Digha Mohona region. Z1, Z2, Z3, and Z4 denote the leaf, soil, and water sampling sites. Source: Google Earth, © Google, Data SIO, NOAA, U.S. Navy, NGA, GEBCO

**Figure 2:**
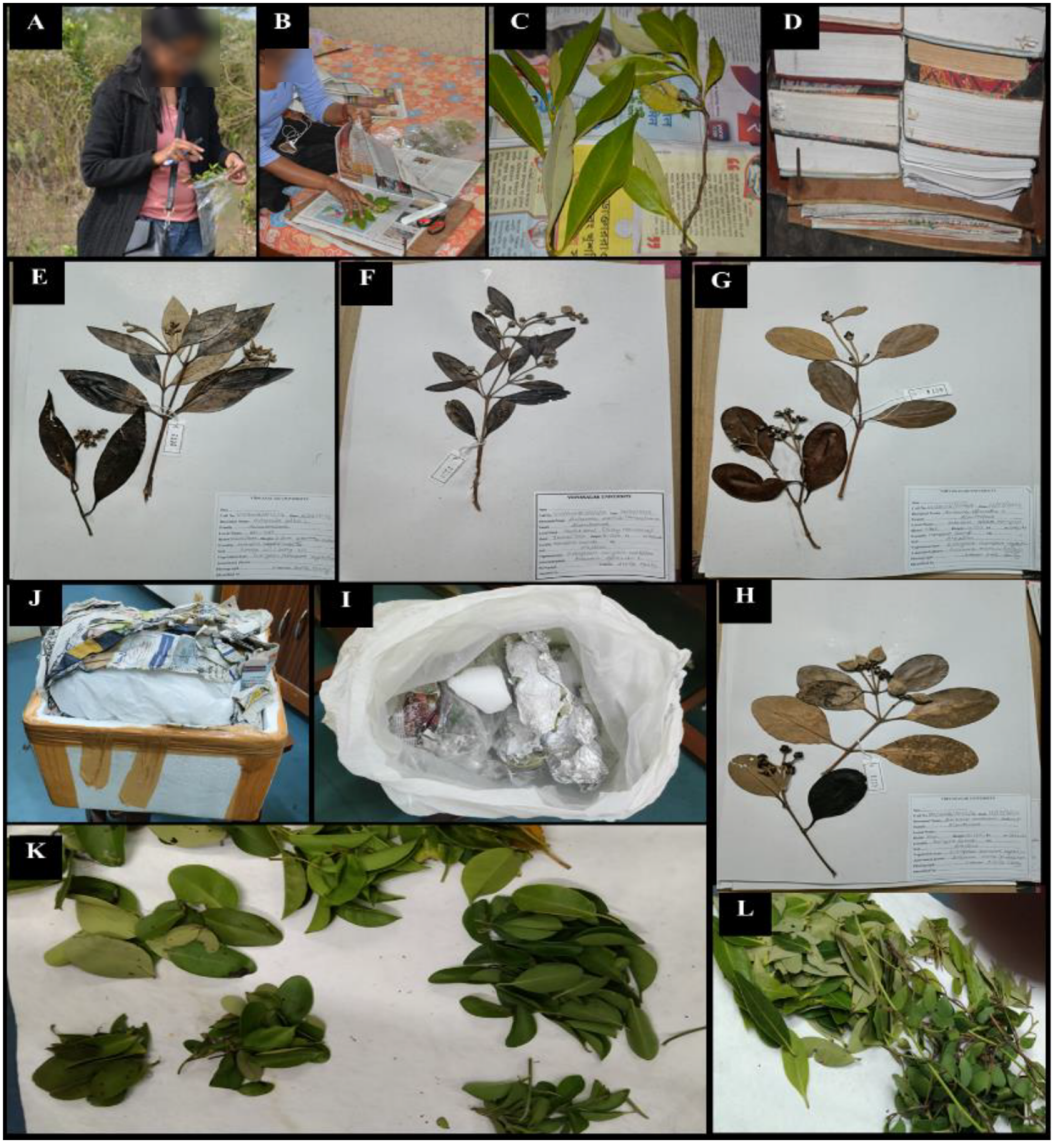
Leaf samples were collected monthly throughout 2023 **(A–D)**; portions were prepared for herbarium specimens **(E–H)**, including *Avicennia alba*, *A. marina*, *A. officinalis*, and *A. rumphiana* **(I, J)**. Fresh leaves were preserved in dry ice **(K, L)** and shade-dried for chemical analysis.

The mentioned plant species were collected from Digha Mohona coastal vegetation toward Champa River adjoining Bay of Bengal located in Ramnagar CD Block I of district Purba Medinipur Coast, West Bengal (**Figure 1**). The longitudinal and latitudinal extension of this area is 87°30′26.7516″ E and 21°37′35.8212″ N, respectively, which has a generally saline loam soil with high pH. Soil and water samples were collected from selected coastal vegetation in the middle of the day on a per-month basis in the year 2023 (**Figure 2**). The geographic coordinates of the collected plant specimens *Avicennia marina*, *Avicennia alba*, *Avicennia officinalis*, and *Avicennia marina* var. *rumphiana* (*A. rumphiana*) were recorded as 21°38.414′ N, 87°33.544′ E; 21°38.407′ N, 87°33.547′ E; 21°38.409′ N, 87°33.557′ E; and 21°38.426′ N, 87°33.557′ E, respectively. The whole 1 year (2023) was divided in three seasons: the first one is monsoon (July–October), the second one is post-monsoon (November–February), and the third one is pre-monsoon (March–June).

**Figure 3:**
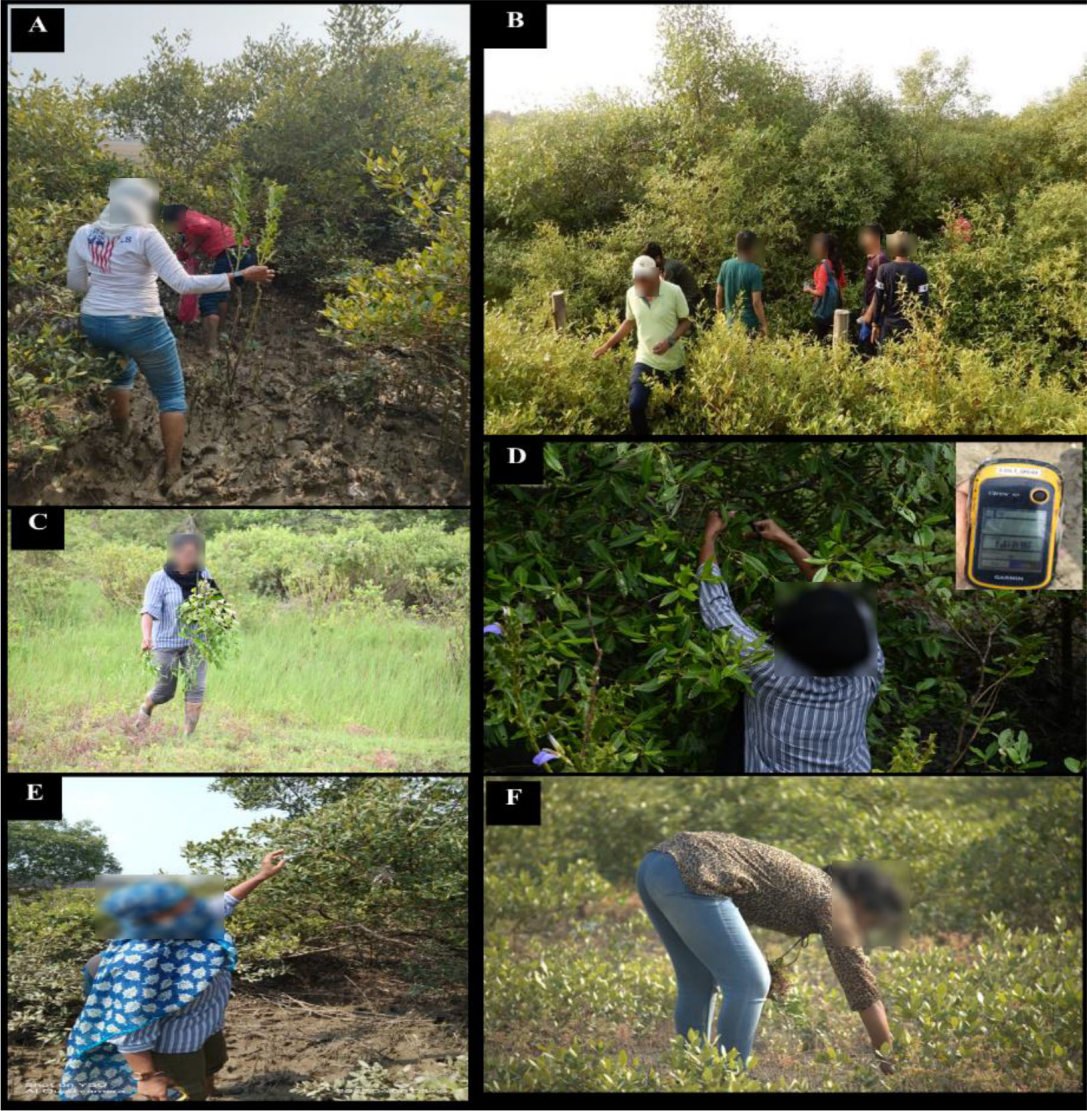
Leaf samples were collected monthly throughout 2023 **(A–F)** from four zones surrounding the Champa River site, with leaves harvested from three replicate trees of each species.

**Figure 4:**
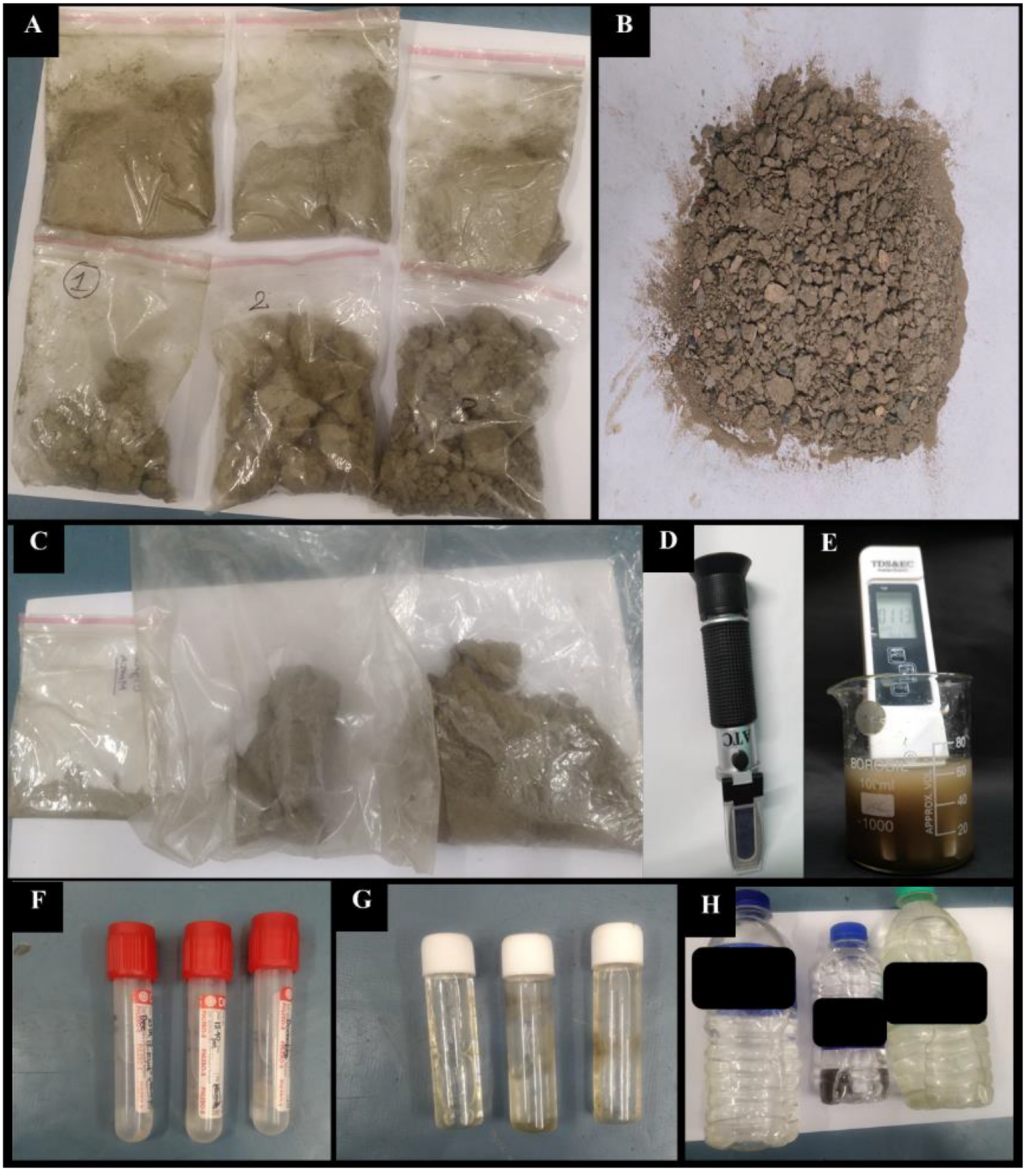
Soil and water samples were collected monthly throughout 2023 from three zones surrounding the Champa River site **(A–C)**. The soil samples are shown in **(D, E)**, while salinity, EC, and TDS were measured using a refractometer and EC/TDS meter. **(F–H)** Water samples.

The sampling sites were selected based on plant availability and their distance from the Digha Mohona river channel. The stretch from the inner boundary of the coastal vegetation to Mohona (~4 km) was divided into four zones (Z1–Z4); a total of 12 sampling (leaf) sites were established according to river proximity and the distribution of four *Avicennia* species (**Figure 3**). Sites DH3 and DH4 were the farthest from Mohona, whereas DH1 and DH2 were the closest to Mohona. *Avicennia marina* and *A. alba* were widely distributed across all zones, allowing four replicate sites per zone; however, *A. rumphiana* occurred only in Z3 and Z4, and *A. officinalis* was present in Z1, Z3, and Z4 but absent in Z2. At each site, three replicate subplots spaced 100–200 m apart were established. Fresh leaves harvested on the 25th of each month throughout 2023 from branches less than 2 ft. above the ground level and from herbarium specimens (15–20-cm stem tips) were sampled. Leaf sampling came from three replicates of each of the four *Avicennia* species across all zones. The samples were preserved in dry ice and transported to the laboratory (**Figure 2**). The soil samples were collected from four selected zones (Z1, Z2, Z3, and Z4) and stored in zipper bags from each zone; meanwhile, the water samples were collected in tubes for immediate measurement of salinity, electrical conductivity (EC), temperature, and total dissolved solids (TDS), with additional samples preserved in collection bottles for further analysis (**Figure 4**).

### 2.2 Leaf micro-morphological studies

After collection of the mangrove leaves, these were preserved in 70% alcohol or in FAA solution for micro-morphological analysis. The leaves were soaked on 3% KOH and heated for 5 min. Then, the epidermis layer was peeled off and stained with safranin. The stained epidermis was observed using a phase contrast microscope (Zeiss Primostar). The salt gland index of the leaf epidermis was determine by using the following formula:

Salt gland index (SGI) = {number of salt glands in a given area (S)/total number of cells in the area} × 100.

### 2.3 Leaf succulence (relative water content, RWC) measurement

RWC serves as a valuable metric for assessing a plant’s hydration level and its response to drought-induced water stress (Mayak et al., 2004a). This method effectively indicates the water content in plant tissues relative to their maximum water-holding capacity (Ahmed et al., 2021). RWC is an appropriate estimate of plant cellular hydration under the effect of both leaf osmotic adjustment and water potential. The measurement involves collecting mature leaves and cutting them into pieces (2 cm × 2 cm), which gives their fresh weight. To find the turgid weight, the leaf samples were hydrated in tri-distilled water at 4°C in the dark for 6 h. The samples were dried in a hot air oven at 75°C for 48 h to obtain the dry weight.

The RWC is calculated using the formula followed by Weatherley (1950). FW means fresh weight, DW means dry weight, and TW means turgid weight.


RWC=(FW–DW)/(TW–DW)×100

WSD=(100−RWC)

### 2.4 Compatible osmolytes content

#### 2.4.1 Proline concentration measurement

Proline concentration was determined following the method described by Bates et al. (1973). This technique provides a quantitative colorimetric measurement of the proline content of plants to determine the plant’s physiological condition and evaluate its stress tolerance (Abraham et al., 2010). A 0.5-g sample of frozen leaves was homogenized in 5 mL of 3% (w/v) aqueous sulfosalicylic acid solution. The homogenated extract was centrifuged at 15,000 *g* for 10 min at 4°C. Then, 2 mL of supernatant was mixed with 2 mL of acid ninhydrin (0.125% ninhydrin/30 mL glacial acetic acid/20 mL of 6 M phosphoric acid) and 2 mL of glacial acetic acid. The acid ninhydrin must be prepared fresh and is only stable for 24 h at 4°C. The mixture was maintained in water bath at 100°C for 1 h, and the reaction was stopped in an ice bath. Then, 4 mL of toluene was then added to this solution, and it was stirred for 15–20 s. The upper phase was collected, which was toluene, and the optical density was determined by measuring the absorbance at 520 nm using a spectrophotometer; toluene was used as the control. Known concentrations of known L-proline solutions (for example, 0.0, 20, 40, 60, 80, and 100 ppm/mL) were used in preparing the standard curve of proline.


$$\begin{array}{l} Proline\text{ concentration µmoles proline}/\text{g of fresh weight }(\text{FW})\text{ material}\\ =[(\text{μg proline}/\text{ml})\\ \times \text{ml toluene})/115.5\text{μg}/\text{μmole}]/[(\text{g sample})/5] \end{array}$$

#### 2.4.2 Glycine betaine concentration determined by periodide spectrophotometric methods

The method was carried out following Grieve and Grattan, 1983. Five grams of fresh sample, finely ground plant tissue was mixed with 10 mL of dH_2_O for 24 h and then filtered. The 2-mL filtrate was mixed with an equal volume of 2N H_2_SO_4_ (1:1) and cooled over ice. Then, 50 μL of KI-I2 reagent was added to the mixture and gently stirred, and then the mixture was stored at 4°C for 16 h. The mixture was centrifuged at 12,000 *g* for 15 min at 4°C. The precipitated iodine crystals were dissolved in 1.5 mL of 1,2-dichloroethane with a vortex mixer, and absorbance was measured at 365 nm after 2 h. A glycine betaine (GB) solution in 1 M H_2_SO_4_ was used as the standard.

GB was calculated according to the following formula:


$$\begin{array}{l} \text{GB concentration µmoles GB}/\text{g of FW material}\\ =[(\text{μg GB}/\text{ml})\times \text{ml }1,2\\ -\text{dichloroethane})/117.15\text{μg}/\text{μmole}]/(\text{g sample}) \end{array}$$

#### 2.4.3 Sugar alcohol concentration determination by spectrophotometric methods

The method was carried out following Lewis and Harley (1965). A 0.5-g fresh sample was homogenized with 70% cold methanol, and then 1 mL of methanolic extract was diluted with 1 mL of 1 M acetate buffer (pH 4.5). After 15 min, 1 mL of 0.75% sodium metaperiodate solution was added to the solutions. The reaction was activated within 5 min, and the optical density was measured at 260 nm using a UV spectrophotometer (Shimadzu UV-NIR-3600). D-mannitol and D-sorbitol were used as a standard to create a calibration curve, allowing the determination of sugar alcohol concentrations in the samples.

Mannitol and sorbitol were calculated using the following formula:


$$\begin{array}{l} \text{Mannitol concentration µmoles Mannitol}/\text{g of FW material}\\ =[(\text{μg mannitol}/\text{ml})\\ \times \text{ml sodium metaperiodate})/182.172\text{μg}/\text{μmole}]/(\text{g sample}) \end{array}$$

$$\begin{array}{l} \text{Sorbitol concentration µmoles Sorbitol}/\text{g of FW material}\\ =[(\text{μg Sorbitol}/\text{ml})\\ \times \text{ml sodium metaperiodate})/182.17\text{μg}/\text{μmole}]/(\text{g sample}) \end{array}$$

### 2.5 Plant leaf pigments and metabolite concentration determination

#### 2.5.1 Chlorophyll content measurement

The method was carried out following Schlemmer et al. (2013). At first, samples of all plants from a study area were collected in a zipper pouch. Then, 0.5 g of fresh leaves was homogenized with 10 mL of 80% acetone. Then, the acetone solution was centrifuged at 5,000 rpm for 5 min, the supernatant was collected, and the extraction with 80% acetone was repeated until the residue becomes colorless. The final volume was adjusted to 5 mL with 80% acetone, and the absorbance of the solution was measured at 645, 652, and 663 nm using a spectrophotometer (Shimadzu UV-NIR-3600) with 80% acetone as a blank. The chlorophyll content was calculated using the following equations: *A* = absorbance at the specified wavelength, *V* = final volume of chlorophyll extract in 80% acetone, and *W* = fresh weight of the tissue extracted.


$$\begin{array}{l} \text{Mg chlorophyll a}/\text{g tissue}\\ =[12.7\backslash \text{times}\times (\text{A\{}663\})-2.69\backslash \text{times}\times (\text{A\{}645\})\times \text{V}/1000\times \text{W}] \end{array}$$

$$\begin{array}{l} \text{Mg chlorophyll b}/\text{g tissue}\\ =[22.9\backslash \text{times}\times (\text{A}\{645\})-4.68\backslash \text{times}\times (\text{A}\{663\})\times \text{V}/1000\times \text{W}] \end{array}$$

$$\begin{array}{l} \text{Mg total chlorophyll}/\text{g tissue}\\ =[20.2\backslash \text{times}\times (\text{A}\{645\})-8.02\backslash \text{times}\times (\text{A}\_\{663\})\backslash \text{times}\\ \times \text{V}/1000\backslash \text{times}\times \text{W}] \end{array}$$

#### 2.5.2 Soluble carbohydrate measurement

The method was carried out following Nielsen (2009). Total carbohydrate concentration in plant samples was determined using the phenol–sulfuric acid method (Nielsen, 2009). Fresh plant samples (leaf and stem separately) weighing 0.5 g were hydrolyzed with 10 mL of 2.5 N HCL in a boiling water bath for 3 h to break down the complex carbohydrate to simple sugar. The solution was neutralized with solid sodium carbonate (Na_2_CO_3_) to stop the reaction. The effervescence observed was due to the release of CO_2_ during neutralization. The solution was centrifuged at 10,000 rpm for 10 min. Then, 500 μL of supernatant was mixed with 500 µL DH_2_O, 1 mL of 5% phenol, and 500 mL of 98% concentrated H_2_SO_4_. The solution was incubated in a water bath at 25°C–30°C for 20 minutes, and the final color was developed. The optical density was measured at 490-nm wavelength using a UV spectrophotometer (Shimadzu UV-NIR-3600). The standard solution D-glucose was used for standard curve. The carbohydrate content was calculated using the following equations: *B* = amount of total carbohydrate determined from the standard carve (glucose equivalent), *V* = sample volume added into the reaction well, and *M* (g) = mass of the fresh leaf extract used in preparing the solution (in grams).


$$\begin{array}{l} \text{Total Carbohydrate concentration }(\text{mg}/\text{g FW})\\ =[(\text{B}/\text{V})\times \text{Dilution factor}]/\text{M} \end{array}$$

#### 2.5.3 Total phenolic content

Leaf total phenolic content was determined according to the Folin–Ciocalteu method, described by Singleton et al. (1999). The 0.5-mL Folin–Ciocalteu (FC) reagent (1:1) was supplemented to 100 µL of methanolic extract diluted with 900 μL dH_2_O. The reaction mixture was incubated for 40 min at room temperature in the dark. Then, absorbance was measured at 765 nm versus the blank. The difference in concentration of gallic acid (mg/GAE) was used as a standard to create a standard curve. The total phenolic content in the samples was expressed as milligrams of gallic acid equivalent per gram dry weight (mg GAE/g DW). The TPC was calculated using the following equations: *C* (mg/mL) = concentration of GA determined from the standard curve, *V* (mL) = extract volume used in the measurement, and *M* (g) = mass of the dry extract used to prepare the solution (in grams).


TPC (mg/g DW)=(C×V)/M

#### 2.5.4 Determination of total flavonoid content

Total flavonoid estimation was done following the aluminum chloride (AlCl_3_) and sodium acetate (CHCOONa) calorimeter methods (Fernandes et al., 2012). Then, 2 mg of dry extract was mixed with 4 mL of 80% methanol. The solution was centrifuged (8,000 *g* for 5 min), and 0.5 mL of supernatant was diluted with 2 mL of methanol, 100 µL of 7% aluminum chloride (AlCl_3_), and 100 μL of sodium acetate (CHCOONa) and left for 1 h for incubation. After incubation, the mixture’s absorbance at 450 nm was measured against a blank solution by using a UV spectrophotometer (Shimadzu UV-NIR-3600). Quercetin (mg/QE) was used as a standard solution to obtain a standard curve. Total flavonoid concentration in the plant samples was calculated from the standard curve, and the results were expressed as quercetin equivalent per gram dry weight (mg QE/g DW). The TFC was calculated using the following equations: *C* (mg/mL) = concentration of quercetin determined from the standard carve, *V* (mL) = extract volume used in the measurement, and *M* (g) = mass of the dry extract used in preparing the solution (in grams).


TFC (mg/g DW)=(C×V)/M

#### 2.5.5 Determination of total polyphenol content

Total polyphenol were estimated according to the method described by Robert (1971). One milligram of dry powder was dissolved into 1 mL of 90% methanol. Then, 1 mL of vanillin hydrochloride reagent was added, prepared just before use by mixing equal volumes of 8% hydrochloric acid in methanol and 4% vanillin in methanol. Then, using a UV spectrophotometer (Shimadzu UV-NIR-3600), absorbance at 500-nm wavelength was read after 20 min of incubation, using vanillin hydrochloride reagent alone as a blank. Tannic acid (mg/TA) was used as a standard solution to obtain a standard curve. TPPC in the plant samples was calculated from the standard curve, and the results were expressed as tannic acid equivalent per gram dry weight (mg TA/g DW). The TPPC was calculated using the following equations: *C* (mg/mL) = concentration of tannic acid determined from the standard carve, *V* (mL) = extract volume used in the measurement, and *M* (g) = mass of the dry extract used in preparing the solution (in grams).


TPPC (mg/g DW)=(C×V)/M

### 2.6 Statistical analysis

All quantitative data including plant stress tolerance biochemical marker and soil and surface water parameter were statistically analyzed using GraphPad prism v.10. One-way analysis of variance followed by *post hoc* multiple mean comparisons (Tukey’s test) for each dependent variable was performed. All biochemical data were analyzed independently for each species. Additionally, principal component analysis (PCA) of the complete dataset was carried out using PAST. PC1 and PC2 captured most of the observed variability in biplot. Significant covariance among parameters was identified using Pearson correlation coefficient (*r*) (*P* < 0.05) in correlation matrices.

## 3 Results

### 3.1 Seasonal variation effects on plants’ phenological pattern

The flowering, fruiting, and germination periods of *Avicennia* species in Digha Mohona coastal vegetation are summarized in **Table 1**. The *Avicennia* species generally flower throughout the year, with the monsoon and pre-monsoon season having the most blooms. The flowering duration of *Avicennia* sp. varied greatly, lasting from 1 to 6 months. *A. marina* and *A. alba* species exhibited a different reproductive strategy from two other species *A. rumphiana* and *A. officinalis*. Moreover, species like *A. alba* and *A. marina* have flowering duration in the pre-monsoon to monsoon season, while *A. officinalis* and *A. rumphiana* have flowering periods in late pre-monsoon and monsoon months. In Digha coast, *Avicennia* sp. primarily produced mature propagules during the monsoon (August–October) period and germination occurred mostly in the post-monsoon season (November–December).

**Table 1:** Phenology of the four selected mangrove species during the study period in 2023.

Scientific name	Family	Flowering season	Fruiting season	Germination season
*A. alba*	Acanthaceae	May–Aug	Aug–Oct	Nov–Dec
*A. officinalis*		July–Aug	Aug–Oct	Nov–Dec
*A. marina*		May–Aug	Aug–Oct	Nov–Dec
*A. rumphiana*		July–Aug	Aug–Oct	Nov–Dec

### 3.2 Seasonal effects on plant micro-morphology

**Figure 5:**
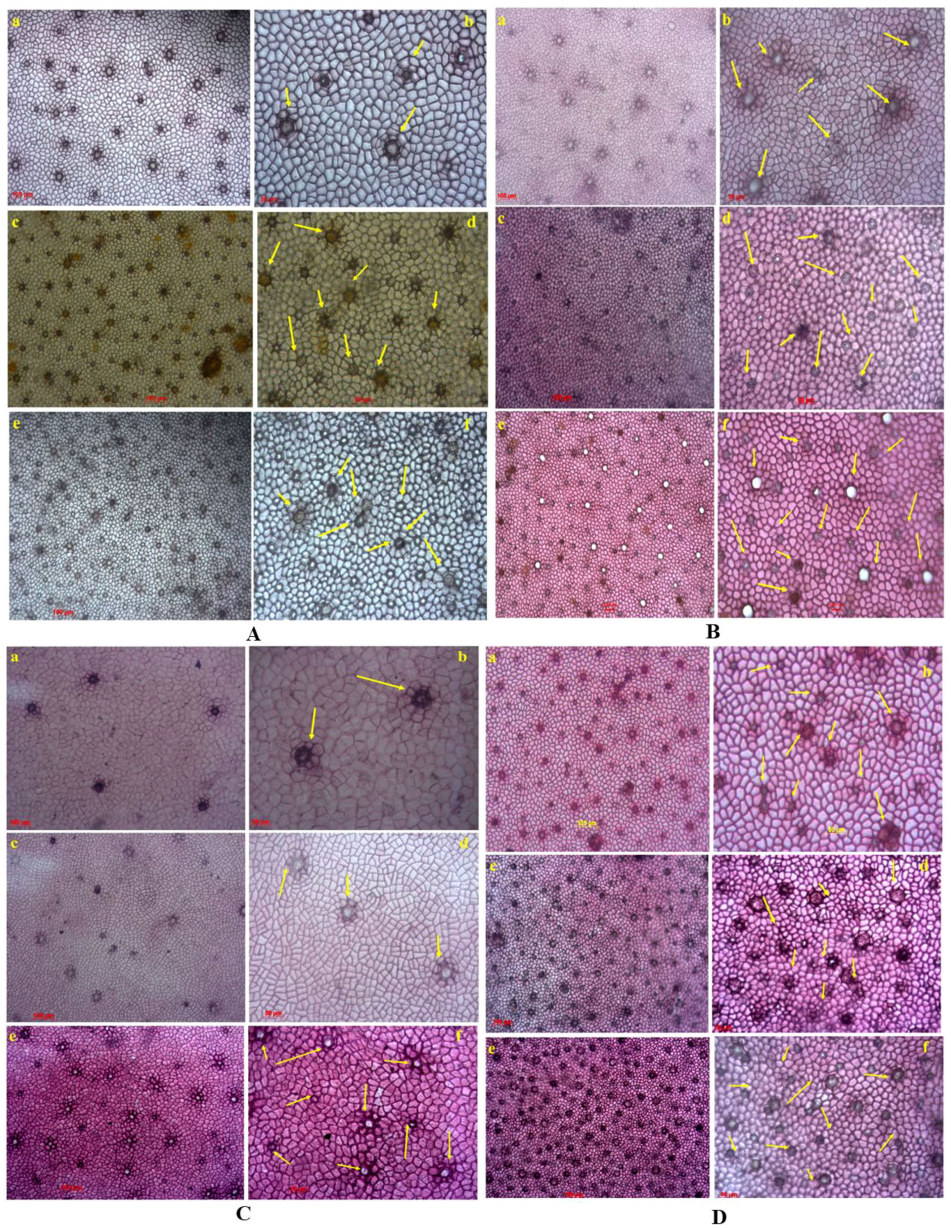
Seasonal changes’ effects on the number of salt glands of the adaxial leaf epidermis of four *Avicennia* sp. **(A)** *A. rumphiana* (a, b represent the salt gland in monsoon season; c, d show the salt gland in post-monsoon season; e, f represent the salt gland in pre-monsoon season). **(B)** *A. marina* (a, b represent the salt gland in monsoon season; c, d showing the salt gland in post-monsoon season; e, f represent the salt gland in pre-monsoon season.) **(C)** *A. alba* (a, b represent the salt gland in monsoon season; c, d show the salt gland in post-monsoon season; e, f represent the salt gland in pre-monsoon season). **(D)** *A. officinalis* (a, b represent the salt gland in monsoon season; c, d showing the salt gland in post-monsoon season; e, f represent the salt gland in pre-monsoon season). Salt glands were observed by using a phase contrast microscope (Zeiss primo star (×10 and ×20 lens). The yellow arrow represents the presence of salt glands.

We set out to investigate the effects of seasonality on physiological adaptation. We observed the number of salt glands as there might be changes in their salt stress level during different seasons. The highest salt gland index (SGI) was found in pre-monsoon season and high salt stress environmental conditions compared with the other two seasons (**Table 2**). As shown in **Figures 5A, B, D**, *A. rumphiana*, *A. marina*, and *A. officinalis* comparatively have the highest salt gland density in post-monsoon and pre-monsoon seasons. Generally, the lowest salt gland density was found in *A. alba* during all seasons (**Figure 5C**). In **Table 2**, the highest SGI was present in *A. rumphiana* (4.28%) and *A. officinalis* (3.75%) leaf epidermis during the pre-monsoon season and the lowest in *A. alba* (0.23%) during the monsoon season. Moreover, we observed that the salt glands formed only in the post- and pre-monsoon seasons for the removal of excess salt from cellular levels.

**Table 2:** Salt gland index of selected *Avicennia* sp. leaf epidermis.

Plant name	Number of salt gland per unit area			Number of epidermal cell per unit area			Salt gland index (%)		
	Monsoon	Post-monsoon	Pre-monsoon	Monsoon	Post-monsoon	Pre-monsoon	Monsoon	Post-monsoon	Pre-monsoon
*A. rumphiana*	8 ± 1.5	31 ± 2	48 ± 3	822 ± 9	954 ± 6.7	1,073 ± 7.6	1	3.1	4.3
*A. marina*	10 ± 1	30 ± 1.5	41 ± 2	1,154 ± 6.6	1,359 ± 5.2	1,334 ± 6.1	0.9	2.1	3
*A. alba*	2 ± 1	3 ± 1	10 ± 2	881 ± 7	1,121 ± 6.1	971 ± 5.6	0.2	0.3	1
*A. officinalis*	25 ± 1.5	40 ± 2.5	50 ± 2	1,032 ± 9.1	1,127 ± 7	1,275 ± 5.7	2.3	3.5	3.8

### 3.3 Environmental factors and soil condition changes in different seasons

**Figure 6:**
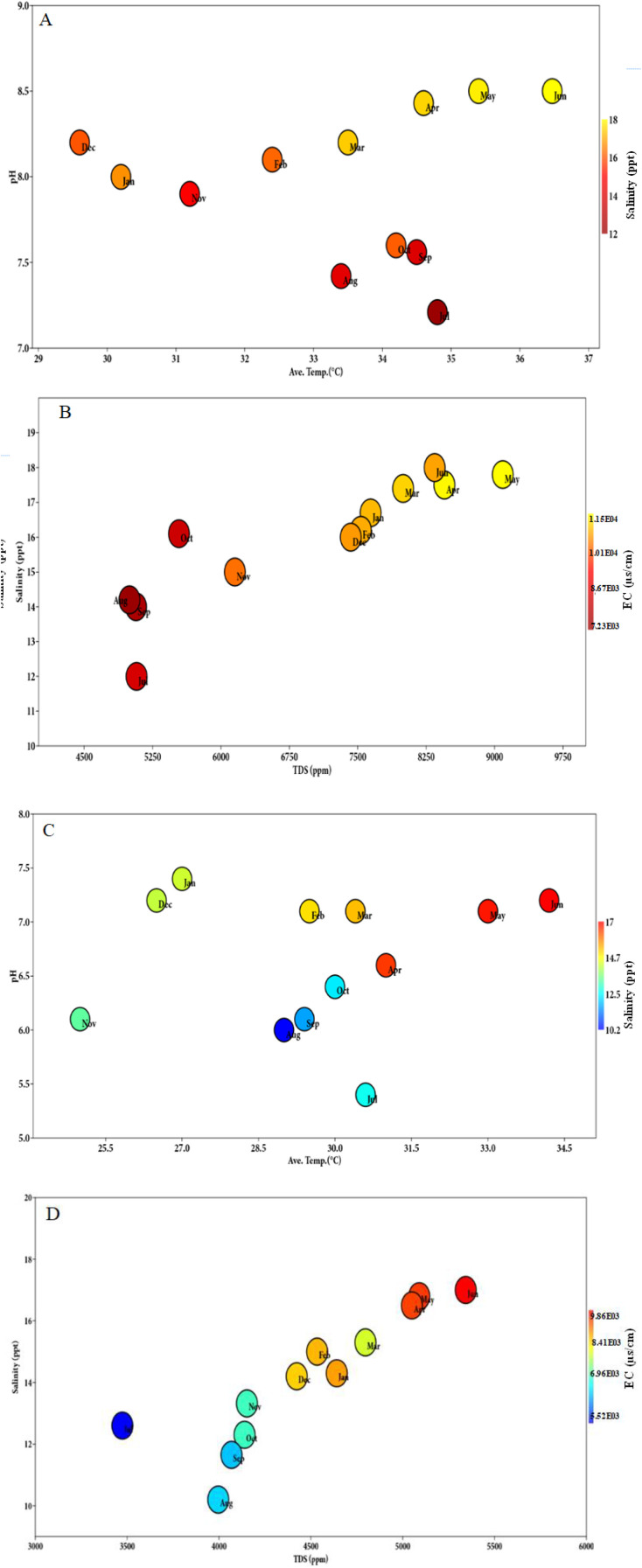
**(A)** Bubble graph representing the monthly variation in average temperature, pH, and salinity of surface water during the study year 2023. **(B)** Monthly variation in salinity, TDS, and EC recorded during the study period. **(C)** Bubble graph representing the monthly variation in soil average temperature, pH, and salinity during the study year 2023. **(D)** Monthly variation in salinity, TDS, and EC recorded during the study period.

Environmental parameters recorded from soil and surface water are depicted in **Tables 3**, **4**. A strong seasonal trend of environmental parameters was observed throughout the study year 2023. Salinity and pH were significantly lower during the monsoon period than during pre-monsoon and post-monsoon in both soil and surface water (*P* < 0.05). TDS and EC were significantly higher in pre-monsoon compared to post-monsoon (two-way ANOVA, *P* < 0.05). During the pre-monsoon season, salinity and temperature were relatively higher, accompanied by increased pH and electrical conductivity in both soil and surface water. High pH affects their conductivity and total dissolved solids. During the monsoon season, rainwater and surface runoff typically decrease the pH, salinity, and TDS in both surface water and soil due to the dilution effect. However, electrical conductivity was closely correlated with TDS (**Figure 6**).

**Table 3:** Monthly variation in average temperature, pH, salinity (ppt), EC (µs/cm), and TDS (ppm) of water surface during study period.

Month	Season	Ave. temp. (°C)	pH	Salinity (ppt)	TDS (ppm)	EC (µs/cm)
Jan	Post-monsoon	30.2	8	16.7	7,641	10,956
Feb	Post-monsoon	32.4	8.1	16.2	7,534	10,823
Mar	Pre-monsoon	33.5	8.2	17.4	7,997	11,161
Apr	Pre-monsoon	34.6	8.43	17.5	8,450	11,484
may	Pre-monsoon	35.4	8.5	17.8	9,090	11,536
Jun	Pre-monsoon	36.47	8.5	18	8,343	10,761
Jul	Monsoon	34.8	7.21	12	5,074	8,515
Aug	Monsoon	33.4	7.42	14.2	4,996	7,232
Sep	Monsoon	34.5	7.56	14	5,068	7,624
Oct	Monsoon	34.2	7.6	16.1	5,539	8,314
Nov	Post-monsoon	31.2	7.9	15	6,152	10,332
Dec	Post-monsoon	29.6	8.2	16	7,423	10,679

**Table 4:** Monthly variation in average temperature, pH, salinity (ppt), EC (µs/cm), and TDS (ppm) of soil during the study period.

Month	Season	Ave. temp. (°C)	pH	Salinity (ppt)	TDS (ppm)	EC (µs/cm)
Jan	Post-monsoon	27	7.4	14.3	4,641	8,956
Feb	Post-monsoon	29.5	7.1	15	4,534	8,823
Mar	Pre-monsoon	30.4	7.1	15.3	4,797	8,161
Apr	Pre-monsoon	31	6.6	16.5	5,050	9,484
May	Pre-monsoon	33	7.1	16.8	5,090	9,536
Jun	Pre-monsoon	34.2	7.2	17	5,343	9,861
Jul	Monsoon	30.6	5.4	12.6	3,474	5,515
Aug	Monsoon	29	6	10.2	3,996	6,732
Sep	Monsoon	29.4	6.1	11.65	4,068	6,624
Oct	Monsoon	30	6.4	12.3	4,139	7,314
Nov	Post-monsoon	25	6.1	13.32	4,152	7,332
Dec	Post-monsoon	26.5	7.2	14.2	4,423	8,679

### 3.4 Seasonal variation effects on plants’ leaf pigments

**Figure 7:**
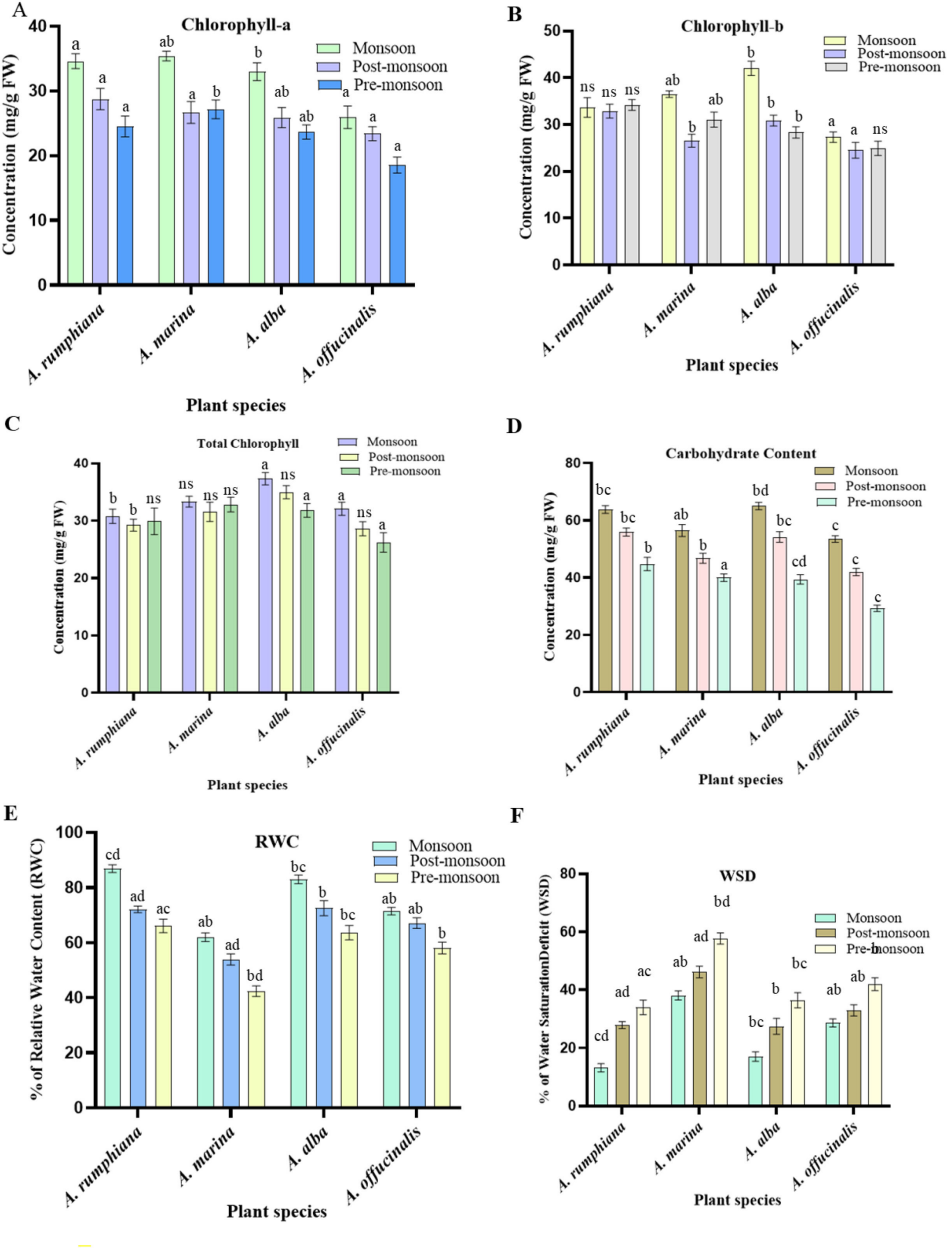
Change in leaves’ pigment, succulence, and carbohydrate production of four *Avicennia* sp. under different seasons. **(A)** Chlorophyll-a, **(B)** chlorophyll-b, **(C)** total chlorophyll, **(D)** carbohydrate contents (CABC), **(E)** relative water content (RWC), and **(F)** water saturation deficit (WSD) in different seasons. Data are shown as mean ± SE. Means followed by different letters indicate a significant difference (*p* < 0.05).

During the pre-monsoon seasons, plants typically suffered from drought, high temperature, and salinity that affect chlorophyll production and thus photosynthesis in all *Avicennia* sp. In post-monsoon seasons, lower temperatures and shorter daylight hours resulted in senescence and reduced chlorophyll content. Conversely, the monsoon seasons provided high water availability, which helped in the higher production of chlorophyll and prevented chlorophyll degradation; adequate water supply promotes the total production of chlorophyll in *A. alba* (37.3 mg/g FW), followed by *A. marina* (33.3 mg/g FW), *A. officinalis* (32.1 mg/g FW), and *A. rumphiana* (30.8 mg/g FW), which was significant at 0.05 levels. Although the Digha Mohona mangrove vegetation is in a tropical region with minimal seasonal temperature variations, chlorophyll production was relatively stable throughout the year (**Figures 7A–C**).

### 3.5 Seasonal variation effects on leaf carbohydrate production

Shoot soluble carbohydrate contents were increased from monsoon to post-monsoon in all plants. The maximum contents of soluble carbohydrates during the monsoon season was significant at 0.05 levels. *A. alba* shoot recorded the highest levels of soluble carbohydrates (65 mg/g FW) in the monsoon season, whereas *A. officinalis* shoot had the lowest concentration of carbohydrate (29.3 mg/g FW) in the pre-monsoon season (**Figure 7D**).

### 3.6 Seasonal variation effects on plants leaf water content

**Figure 8:**
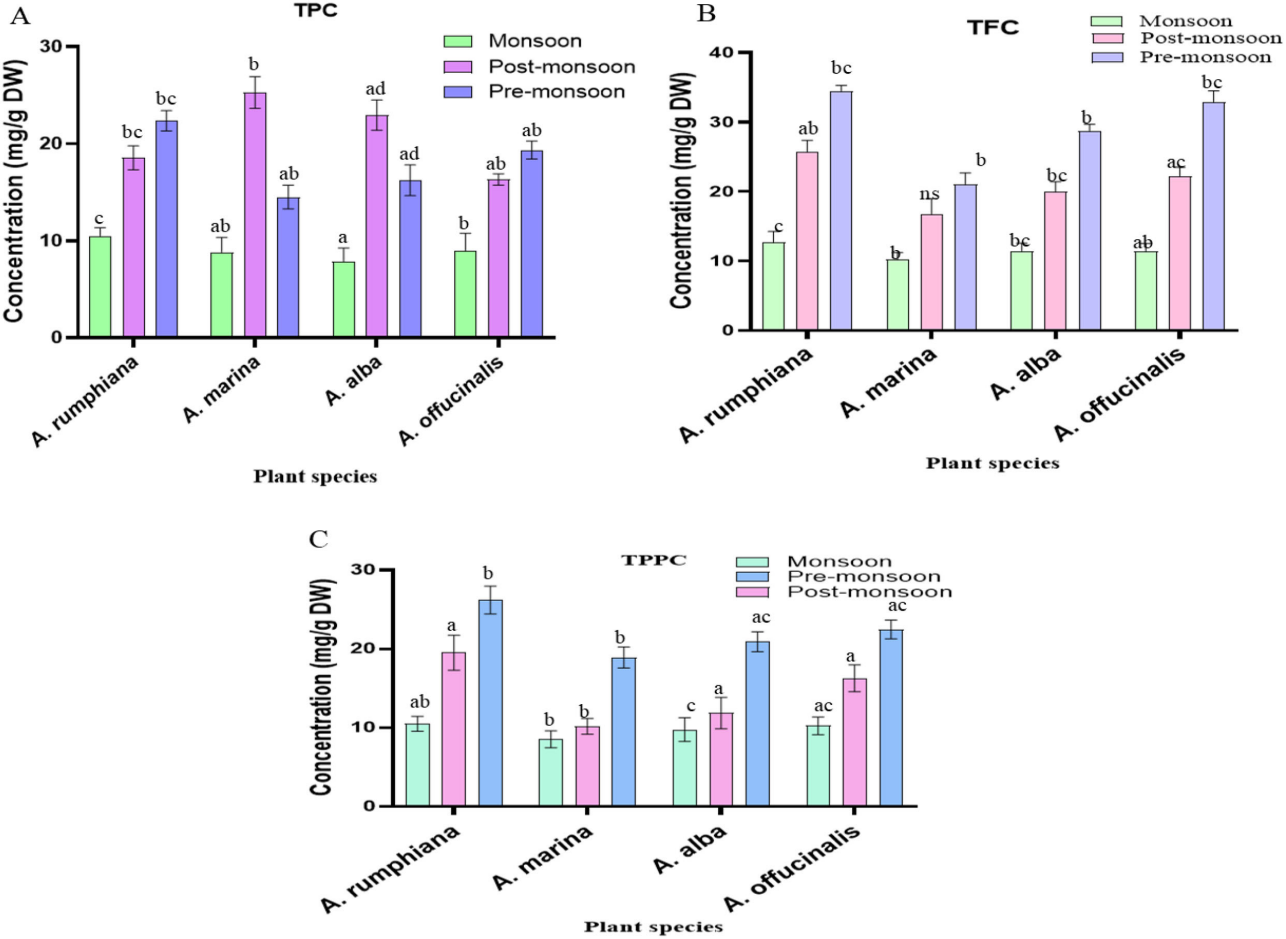
Changes in the secondary metabolites in the leaves of four *Avicennia* sp. **(A)** Total phenol content (TPC), **(B)** total flavonoids content (TFC), and **(C)** total polyphenol content (TPPC). Data are shown as mean ± SE. Means followed by different letters indicate a significant difference (*p* < 0.05). AR, *A. rumphiana*; AO, *A. officinalis*; AB, *A. alba*; AM, *A. marina*.

The mangrove leaves’ relative water content (RWC) and biochemical production are significantly affected by seasonal changes. **Table 5** shows that during pre-monsoon and late post-monsoon seasons, the mangrove leaves tend to have lower relative water content and higher WSD due to water stress (**Figures 7E, F**), which can lead to an increased production of biochemicals like phenols, flavonoids, and polyphenols which protect against oxidative stress. In contrast, the monsoon and early post-monsoon seasons bring higher water availability, resulting in higher relative water content in *A. alba* (83% and 72.5%) and *A. rumphiana* (86.8% and 72.1%). A higher RWC increases chlorophyll production and reduces chlorophyll degradation. The negative correlation between leaf relative water content and biochemical production varies among mangrove species (**Figure 8**)—for example, *A. rumphiana*, *A. alba*, *A. marina*, and *A. officinalis* exhibit different patterns of chlorophyll-a, chlorophyll-b, and total chlorophyll content in all seasons. *A. rumphiana* exhibits a negative correlation between leaf relative water content and compatible osmolyte production, with increased chlorophyll production during water stress. *A. officinalis* shows the lowest leaf relative water content across seasons, with less pronounced changes in chlorophyll and carbohydrate production.

**Table 5:** Concentration of leaf pigment, soluble sugar (mg/g FW), and leaf succulence (% of RWC and WSD) in the leaves of the halophyte *Avicennia* sp. during pre-monsoon, monsoon, and post-monsoon in the year 2023.

Season	Species	Carb (mg/g)	Chl-a (mg/g)	Chl-b (mg/g)	Total Chl (mg/g)	RWC (%)	WSD (%)
Monsoon	AR	63.8 ± 1.36^bc^	34.6 ± 1.14^a^	33.7 ± 2^ns^	30.8 ± 1.25^b^	86.8 ± 1.4^cd^	13.2 ± 1.4^cd^
	AM	56.5 ± 2^ab^	35.4 ± 0.75^ab^	36.5 ± 0.7^ab^	33.3 ± 1^ns^	61.9 ± 1.6^ab^	38.1 ± 1.6^ab^
	AB	65.0 ± 1.3^bd^	33.0 ± 1.36^b^	42.0 ± 1.5^b^	37.3 ± 1^a^	83.0 ± 1.6^bc^	17.0 ± 1.6^bc^
	AO	53.5 ± 1.18^c^	25.9 ± 1.74^a^	27.3 ± 1.1^a^	32.1 ± 1.1^a^	83.0 ± 1.7^ab^	28.6 ± 1.4^ab^
Post-monsoon	AR	56.0 ± 1.4^bc^	28.8 ± 1.63^a^	32.9 ± 1.5^ns^	29.2 ± 1^b^	83.0 ± 1.8^ad^	27.9 ± 1.2^ad^
	AM	46.8 ± 1.76^b^	26.7 ± 1.7^a^	26.5 ± 1.4^b^	31.6 ± 1.7^ns^	83.0 ± 1.9^ad^	46.1 ± 2^ad^
	AB	54.2 ± 1.87^bc^	25.9 ± 1.5^ab^	30.9 ± 1.2^b^	35.0 ± 1.15^ns^	83.0 ± 1.10^b^	27.5 ± 2.8^b^
	AO	42.0 ± 1.3^c^	23.4 ± 1.1^a^	24.5 ± 1.7^a^	28.6 ± 1.22^ns^	83.0 ± 1.11^ab^	32.9 ± 2^ab^
Pre-monsoon	AR	44.8 ± 2.3^b^	24.5 ± 1.6^a^	34.2 ± 1.15^ns^	29.9 ± 2.3^ns^	83.0 ± 1.12^ac^	34.0 ± 2.5^ac^
	AM	40.0 ± 1.3^a^	27.2 ± 1.4^b^	31.1 ± 1.6^ab^	32.8 ± 1.3^ns^	83.0 ± 1.13^bd^	57.7 ± 2^bd^
	AB	39.4 ± 1.65^cd^	23.7 ± 1.1^ab^	28.3 ± 1.2^b^	31.8 ± 1.2^a^	83.0 ± 1.14^bc^	36.4 ± 2.6^bc^
	AO	29.3 ± 1.14^c^	18.5 ± 1.24^a^	24.9 ± 1.5^ns^	26.3 ± 1.7^a^	83.0 ± 1.15^b^	42.0 ± 2.16^b^

Determined compounds: carbohydrate (Carb), chlorophyll-a (Chl-a), chlorophyll-b (Chl-b), and total chlorophyll (TC). AR, *A. rumphiana*; AO, *A. officinalis*; AB, *A. alba*; AM, *A. marina*). Every trait’s value is the mean ± SE; a significant value is denoted by a different letter, *p* < 0.05.

**Figure 9:**
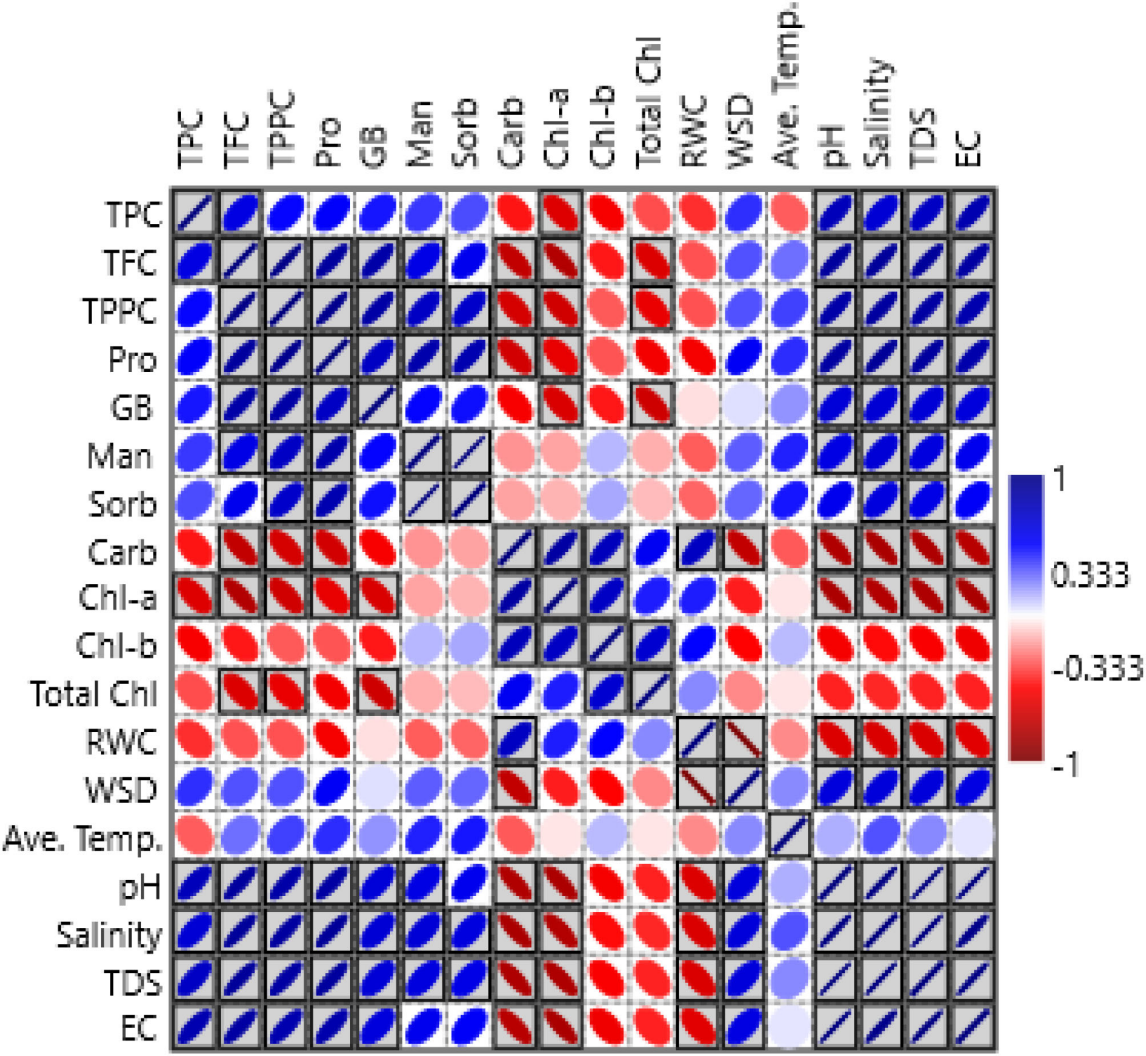
Pearson correlation plot among leaf succulence (% of RWC and WSD), leaf pigments (Chl-a, Chl-b, Total Chl), carbohydrate, compatible osmolytes (Pro, GB, Man, Sorb), and secondary metabolites (TPC, TFC, TPPC) in four different *Avicennia* sp. (AR, AB, AO, and AM) leaves under different seasons (mg g^−1^). The color bar on the right side represents the significant *R*-values (*p* < 0.05).

The mangrove leaves’ relative water content (RWC) and biochemical production were significantly influenced by seasonal variation. As shown in **Table 5**, during the pre-monsoon and late post-monsoon seasons, the mangrove leaves exhibited lower RWC and higher WSD due to water stress (**Figures 7E, F**), which was reflected in the higher production of protective metabolites like phenols, flavonoids, and polyphenols. In contrast, the monsoon and early post-monsoon seasons provided greater water availability, resulting in higher RWC in *A. alba* (83% and 72.5%) and *A. rumphiana* (86.8% and 72.1%). A higher RWC resulted in greater chlorophyll content and reduced chlorophyll degradation. The negative correlation between leaf RWC and biochemical production varied among mangrove species (**Figure 9**), as *A. rumphiana*, *A. alba*, *A. marina*, and *A. officinalis* displayed different patterns of chlorophyll-a, chlorophyll-b, and total chlorophyll content during different seasons. *A. rumphiana* showed a negative correlation between RWC and compatible osmolyte production, with relatively higher chlorophyll content during water stress condition, whereas *A. officinalis* maintained the lowest RWC values during all seasons with comparatively minor changes in chlorophyll and carbohydrate contents.

### 3.7 Effects of seasonal variation on leaf compatible osmolytes

#### 3.7.1 Leaf proline accumulation

**Figure 10:**
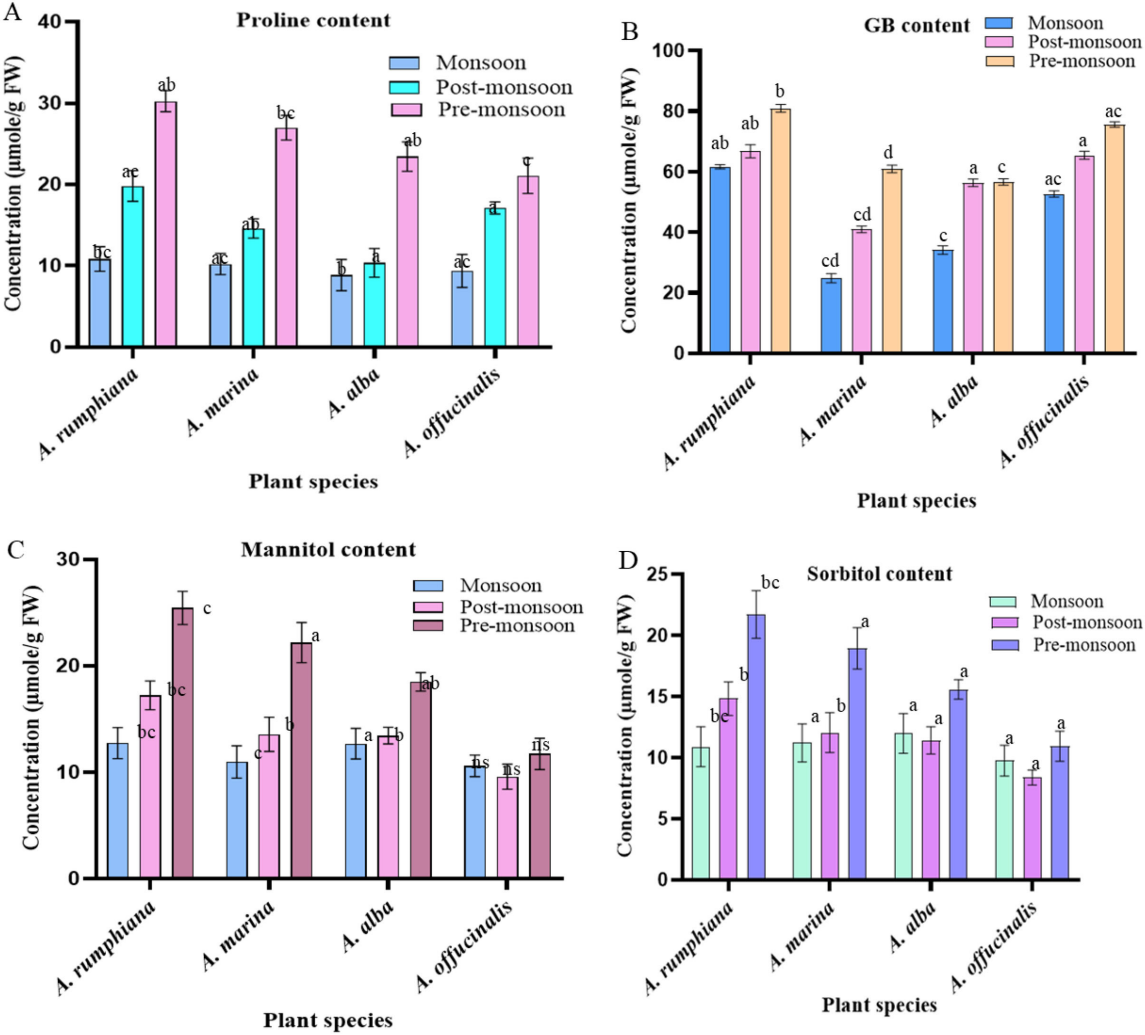
Changes in the compatible osmolyte accumulation of four *Avicennia* sp. under different season. **(A)** Proline content (PC), **(B)** glycine betaine content (GBC), **(C)** mannitol content (MANC), **(D)** sorbitol content (SORC). Data are shown as mean ± SE. Means followed by different letters indicate a significant difference (*p* < 0.05). AR, *A. rumphiana*; AO, *A. officinalis*; AB, *A. alba*; AM, *A. marina*.

Proline accumulation increased in the leaves of all species during post-monsoon and pre-monsoon seasons, with significant changes (*p* < 0.05) in *A. rumphiana*, *A. marina*, and *A. alba*, but not in *A. officinalis.* Higher precipitation during the monsoon season in the Digha Mohona coastal region reduced the soil salinity, as reflected by the lower proline accumulation compared to those of other seasons. During the pre-monsoon period, plants accumulated the highest proline content in *A. rumphiana* and *A. marina*, which was 30.3 μmol/g 0.5 FW and 27.1 μmol/g 0.5 FW, respectively (**Figure 10A**).

#### 3.7.2 Leaf glycine betaine accumulation

The concentration of GB was increased during the post-monsoon and pre-monsoon seasons throughout the study period. The highest accumulation of GB was noted in *A. officinalis* and *A. rumphiana*, which were 80.9 and 75.5 μmol/g 0.5 FW, respectively, in the pre-monsoon season. In contrast, the lowest amount of GB was found in *A. marina* (24.8 μmol/g 0.5 FW) in the monsoon season (**Figure 10B**).

#### 3.7.3 Leaf soluble sugar alcohol accumulation

The sugar alcohol (mannitol) concentration was highest in the leaves of *A. rumphiana* and *A. marina* during the pre-monsoon season of 2023, reaching 25.4 and 22.2 µmol g^-^¹ 0.5 FW, respectively (**Figure 10C**). **Table 6** shows that the sorbitol concentration was also highest in *A. rumphiana* (21.7 μmol/g 0.5 FW) in the pre-monsoon season, and the lowest was recorded in the leaves of *A. officinalis* (8.4 μmol/g 0.5 FW) during the post-monsoon season. Overall, sugar alcohol accumulation showed no significant correlation with environmental factors such as average temperature, EC, TDS, soil salinity, and pH (**Figure 10D**).

**Table 6:** Levels of secondary metabolites and compatible osmolytes (umol g^-1^ dw) in the leaves of the halophyte *Avicennia* sp., during pre-monsoon, monsoon, and post-monsoon in the year 2023.

Season	Species	TPC (mg/g)	TFC (mg/g)	TPPC (mg/g)	Pro (μmol/g)	GB (μmol/g)	Man (μmol/g)	Sorb (μmol/g)
Monsoon	AR	10.5 ± 0.8^c^	12.7 ± 1.56^c^	10.5 ± 0.95^ab^	10.8 ± 1.51^bc^	61.6 ± 0.7^ab^	12.7 ± 1.46^bc^	10.9 ± 1.65^bc^
	AM	8.8 ± 1.53^ab^	10.2 ± 1^b^	8.5 ± 1.06^b^	10.2 ± 1.3^ac^	24.8 ± 1.5^cd^	11.0 ± 1.5^c^	11.2 ± 1.56^a^
	AB	7.9 ± 1.4^a^	11.4 ± 1.2^bc^	9.8 ± 1.5^c^	8.9 ± 1.9^b^	34.1 ± 1.4^c^	12.7 ± 1.4^a^	12.0 ± 1.6^a^
	AO	8.9 ± 1.83^b^	11.4 ± 1.13^ab^	10.2 ± 1.1^ac^	9.4 ± 2^ac^	52.6 ± 1^ac^	10.6 ± 1^ns^	9.8 ± 1.3^a^
Post-monsoon	AR	18.5 ± 1.23^bc^	25.6 ± 1.75^ab^	19.5 ± 2.2^a^	19.8 ± 1.9^ac^	66.7 ± 2.2^ab^	17.2 ± 1.4^bc^	14.8 ± 1.4^b^
	AM	25.3 ± 1.63^b^	16.7 ± 2.29^ns^	10.2 ± 1.02^b^	14.6 ± 1.2^ab^	40.9 ± 1^cd^	13.6 ± 1.6^b^	12.1 ± 1.6^a^
	AB	22.9 ± 1.6^ad^	19.9 ± 1.53^bc^	11.8 ± 2^a^	10.4 ± 1.8^a^	56.3 ± 1.3^a^	13.4 ± 0.78^b^	11.4 ± 1.1^a^
	AO	16.3 ± 0.6^ab^	22.2 ± 1.33^ac^	16.3 ± 1.7^a^	17.1 ± 0.75^a^	65.4 ± 1.3^a^	9.6 ± 1.2^ns^	8.4 ± 0.6^a^
Pre-monsoon	AR	22.4 ± 1.06^bc^	34.4 ± 0.85^bc^	26.2 ± 1.8^b^	30.3 ± 1.3^ab^	80.9 ± 1.3^b^	25.4 ± 1.6^c^	21.7 ± 2^bc^
	AM	14.5 ± 1.21^b^	21.0 ± 1.67^b^	18.9 ± 1.3^b^	27.0 ± 1.5^bc^	60.9 ± 1.2^d^	22.2 ± 1.9^a^	18.9 ± 1.7^a^
	AB	16.2 ± 1.58^ad^	28.7 ± 1^b^	20.9 ± 1.27^ac^	23.4 ± 1.8^ab^	56.6 ± 1^c^	18.5 ± 0.87^ab^	15.6 ± 0.8^a^
	AO	19.3 ± 0.91^ab^	32.9 ± 1.61^bc^	22.5 ± 1.18^ac^	21.1 ± 2.2^c^	75.5 ± 0.87^ac^	11.7 ± 1.5^ns^	10.9 ± 1.23^a^

Determined compounds: total phenol content (TPC), total flavonoids content (TFC), total polyphenol content (TPPC), proline (Pro), glycine betaine (GB), mannitol (man), and sorbitol (Sor). AR, *A. rumphiana*; AO, *A. officinalis*; AB, *A. alba*; AM, *A. marina*). Each value shows the mean ± SE. Significant values are indicated by different letters (*p* < 0.05).

### 3.8 Seasonal effects on leaf secondary metabolites

TPC, TFC, and TPPC were analyzed in four selected *Avicennia* sp. across all seasons in 2023. In the pre-monsoon season, characterized by high salinity and water stress, *A. rumphiana* (22.4 mg GA/g DW) and *A. officinalis* (19.3 mg GA/g DW) accumulated the highest amount of phenolics, especially just prior to flowering (**Figure 8A**). However, *A. marina* (25.3 mg GA/g DW) and *A. alba* (22.9 mg GA/g DW) showed maximum phenol synthesis during the early pre-monsoon (February to April) rosette stage under elevated salinity. In this study, the production of secondary metabolites (TPC, TFC, and TPPC) was lowest at the start of the flowering stage and increased during the fruiting periods with maximum TFC and TPPC from the late post-monsoon to pre-monsoon season (**Table 6**). In contrast, the monsoon season showed the lowest values, including total flavonoids in *A. alba* (7.9 mg QUE/g^-1^ DW) and polyphenol in *A. marina* (8.5 mg TAN/g^-1^ DW) (**Figures 8B, C**). Thus, the seasonal fluctuation in salinity, pH, TDS, and EC influenced the synthesis and accumulation of secondary metabolites.

### 3.9 Principal component analysis and Pearson correlation coefficient analysis

**Figure 11:**
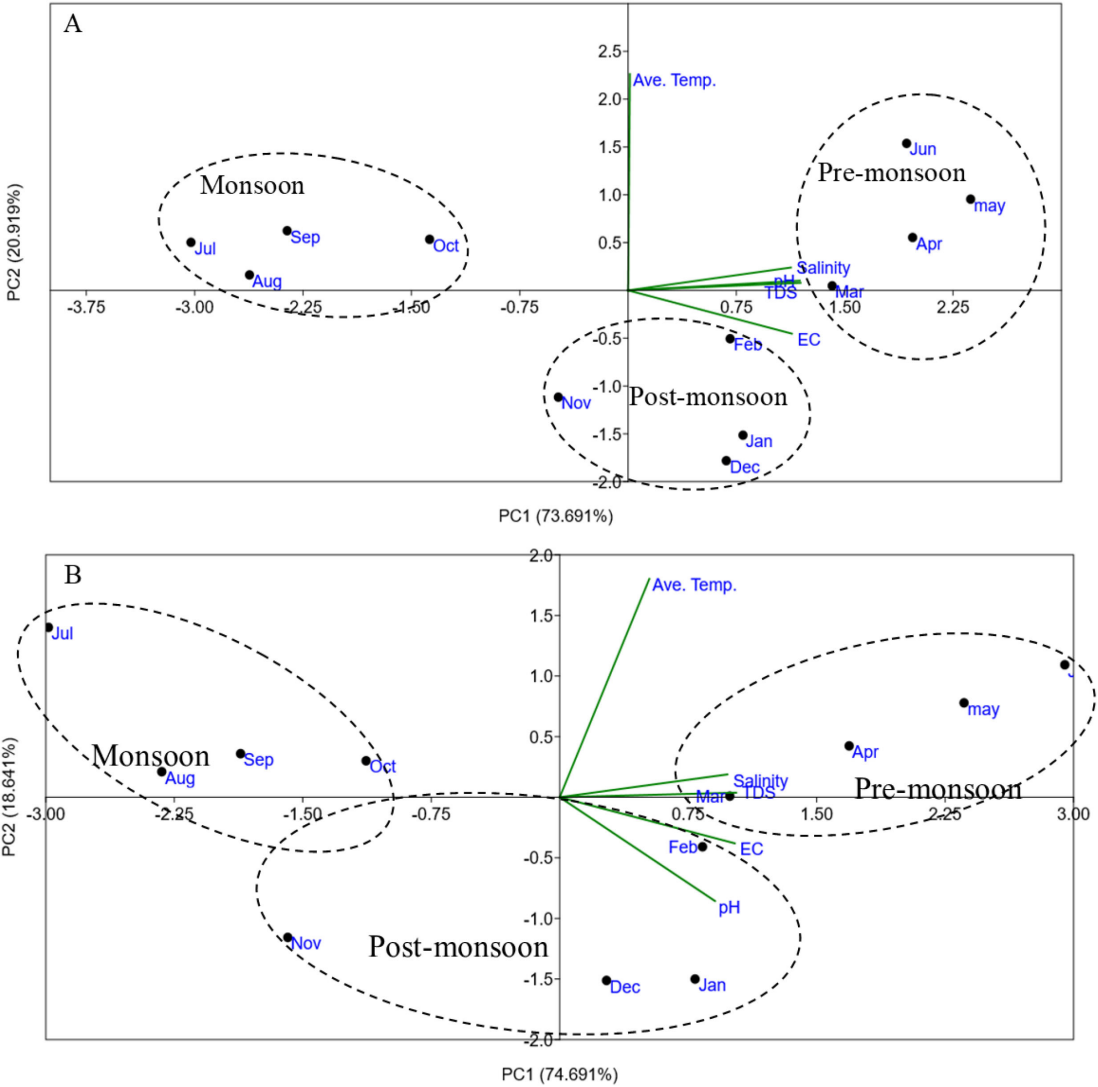
**(A)** Site score plot of the studied variables on the two principal components (PC1, PC2) for monthly environmental parameters of surface water during the study periods. **(B)** Principal component analysis plot of PC1 versus PC2 for the monthly environmental parameters of soil during the study periods.

The principal component analysis (PCA) of soil and surface water parameters showed that PC1 was strongly and positively associated with water and salt stress variables, particularly salinity, pH, EC, and TDS. Seasonal differentiation was evident, as average temperature, pH, salinity, TDS, and EC varied distinctly among monsoon, post-monsoon, and pre-monsoon periods. For surface water, the first two components explained 94.8% of total variation (PC1: 73.89%, PC2: 20.91%), clearly separating the seasons; the pre-monsoon conditions were positively correlated with temperature, pH, salinity, and TDS, whereas EC showed a negative association with the post-monsoon period (**Figure 11A**). Similarly, in soil parameters, the first two components accounted for 90.55% of total variation (PC1: 69.84%, PC2: 20.71%), with temperature, TDS, and EC positively linked to the pre-monsoon season and salinity and pH negatively associated with the post-monsoon season (**Figure 11B**). Overall, most variables loaded positively on PC1, while only temperature showed a positive association and pH, salinity, TDS, and EC showed negative associations on PC2.

**Figure 12:**
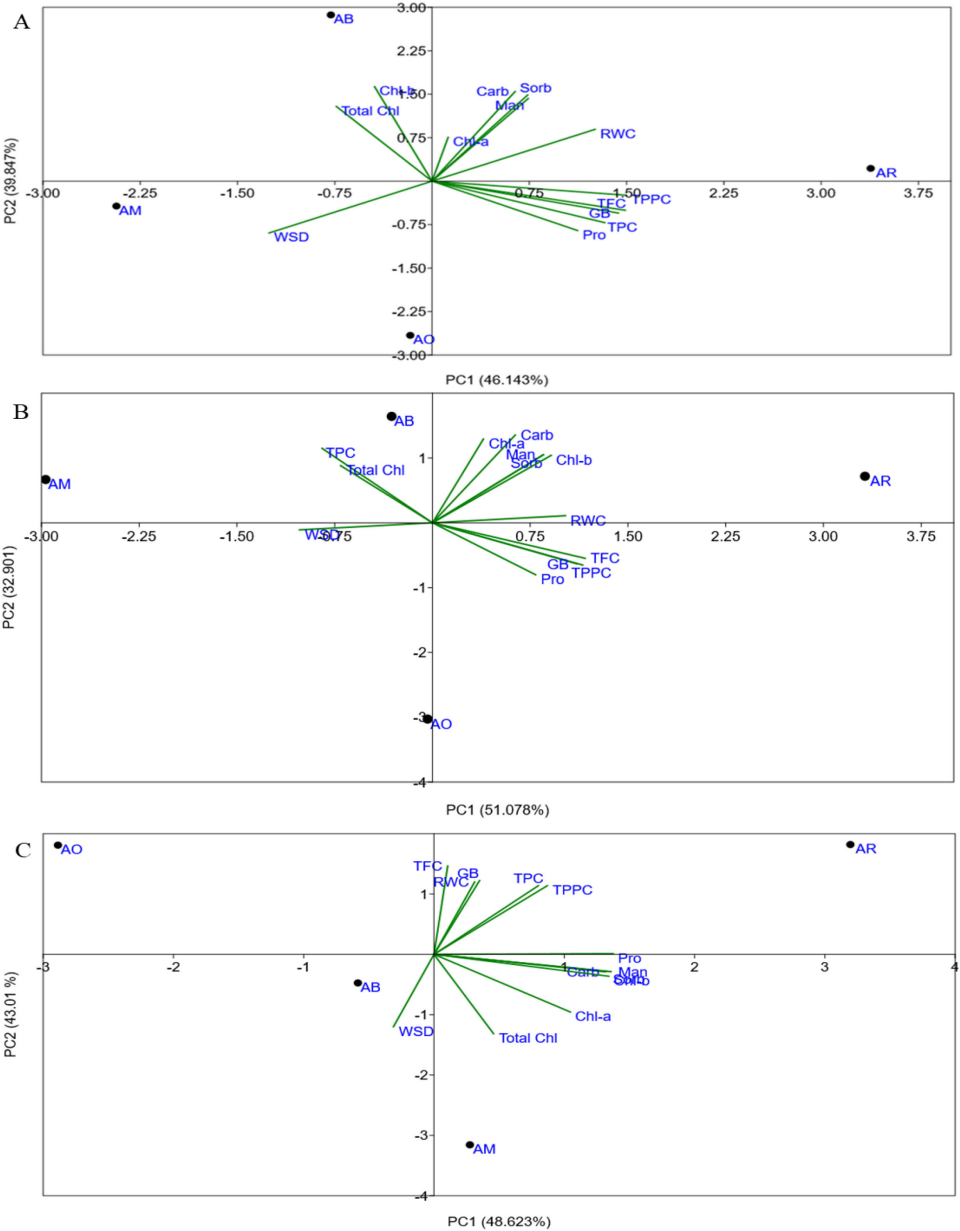
Site score plot of the studied variables on the two principal components (PC1, PC2) for 18 traits among four *Avicennia* spp. under three different seasons. **(A)** Monsoon, **(B)** post-monsoon, **(C)** post-monsoon. Avg. Temp., average temperature; pH; salinity; TDS, total dissolved solid; EC, electrical conductivity; Chl, chlorophyll a, b, total chlorophyll; carb, carbohydrate; RWC, relative water content; WSD, water saturated deficit; Pro, proline; GB, glycine betaine; Mann, mannitol; Sorb, sorbitol; TPC, total phenol content; TFC, total flavonoids content; TPPC, total polyphenol content.

To understand the plant responses, PCA was applied to the physiological and biochemical traits of four *Avicennia* species. The first two principal components cumulatively accounted for more than 70% of the total variance (**Figure 12**). The PCA showed a separation among post-monsoon, summer, and monsoon seasons and different species levels. The secondary molecules (TPC, TFC and TPPC), compatible osmolytes (proline, GB, mannitol, and sorbitol), pigments (Chl-a, Chl-b, and TC), soluble sugar (carbohydrates), and leaf succulence (RWC and WSD) showed species- and season-specific correlations. In general, osmolytes and secondary metabolites were more closely associated with pre-monsoon and post-monsoon stress conditions, while pigments and RWC were associated with monsoon conditions.

The Pearson correlation analysis further examined relationships among morpho-biochemical traits and leaf succulence under varying salinity and water stress conditions (**Figure 9**). The surface water parameters under investigation were temperature, pH, salinity, TDS, and EC. The study focused on the connection between the surface water parameters and the biochemical changes in all seasons. Weak positive correlations were observed between average temperature and pH (*r* = 0.0498), average temperature and salinity (*r* = 0.087), and average temperature and TDS (*r* = 0.052) and negative correlation between average temperature and EC (*r* = -0.165). On the other hand, strong positive correlations were also established between pH and salinity (*r* = 0.92), pH and TDS (*r* = 0.96), pH and EC (*r* = 0.89), salinity and EC (*r* = 0.77), salinity and TDS (*r* = 0.89), and TDS and EC (0.94). Similarly, the soil parameters exhibited a strong positive correlation with salinity and EC (*r* = 0.99), salinity and TDS (*r* = 0.98), pH/EC (*r* = 0.97), and salinity and pH (*r* = 0.91).

In the monsoon season, PC1 and PC2 explained 46.143% and 39.84% of the variation, respectively. PC1 showed strong positive loadings for TPC, TFC, TPPC, Pro, GB, Man, Sor, Carb, Chl-a, and RWC, whereas Chl-b, TC, and WSD were negatively associated. PC2 showed positive correlations with Man, Sor, Carb, Chl-b, TC, and RWC and negative correlations with Pro, GB, TPC, TFC, TPPC, and WSD (**Figure 12A**).

In the post-monsoon season, PC1 and PC2 explained 51.08% and 32.9% of the variation. All biochemical parameters and pigments, including TPC, TFC, TPPC, Pro, GB, Man, Sor, Carb, Chl-a, Chl-b, TC, and RWC, were positively correlated with PC1, while WSD was negatively correlated. PC2 was positively correlated to TPC, TFC, TPPC, proline, GB, and RWC trait but negatively correlated to sugar alcohols, carbohydrates, pigments, and WSD (**Figure 12B**).

In the pre-monsoon season, the cumulative variation was explained by PC1 and PC2, with 48.62% and 43.01% variation, respectively. The positive selections for the pre-monsoon season included TPC, TFC, TPPC, proline, GB, mannitol, sorbitol, carbohydrates, chlorophyll-a, chlorophyll-b, and RWC, while TC and WSD were negatively associated with PC1. In the case of PC2, the variables associated were TPC, mannitol, sorbitol, carbohydrate, Chl-a, Chl-b, TC, and RWC, but it was negatively correlated with proline, GB, TFC, TPPC, and WSD (**Figure 12C**).

**Figure 13:**
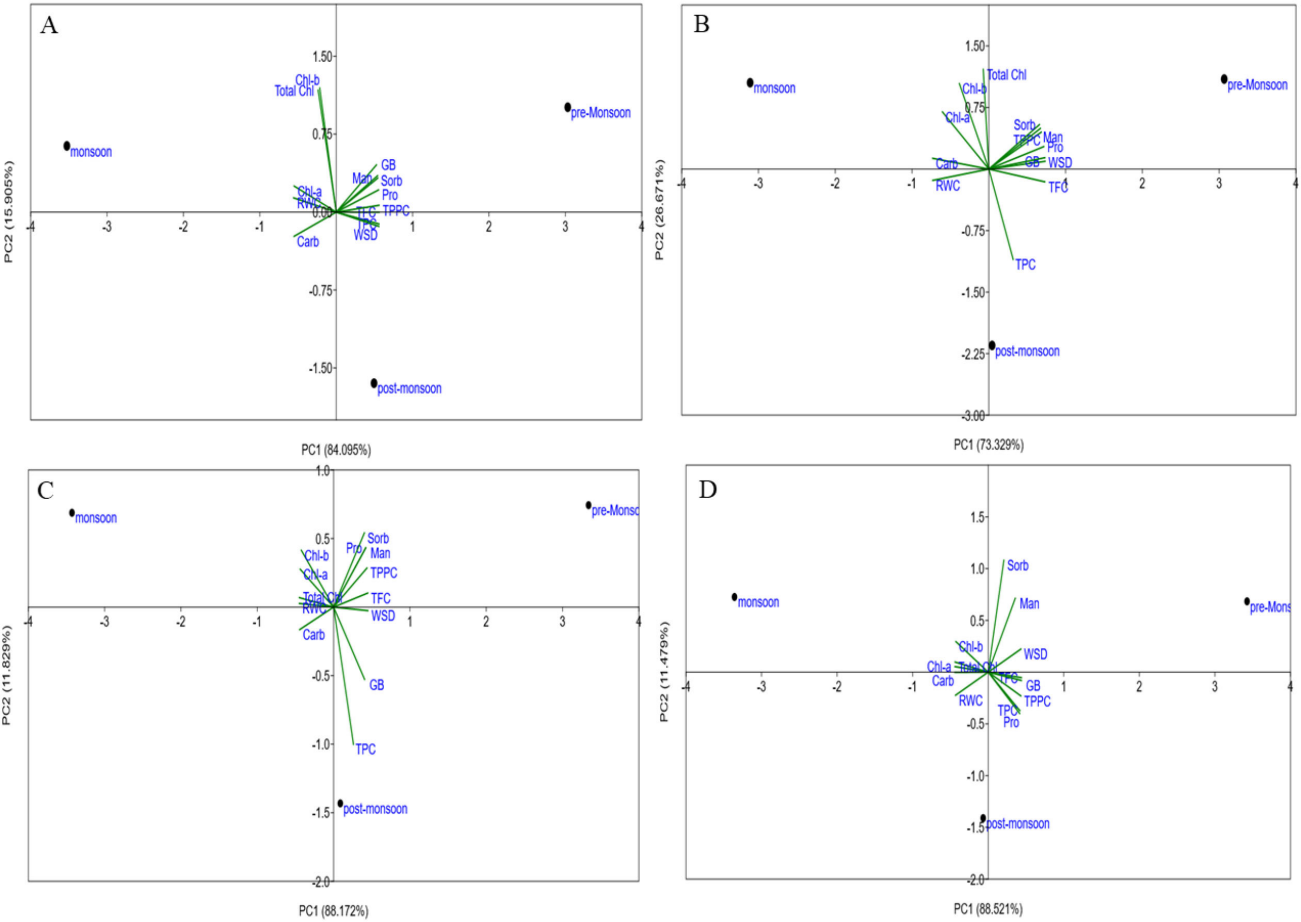
The biplot for the first two principal components was analyzed using the principal component analysis (PCA) for 18 traits among four mangroves *A. rumphiana* **(A)**, *A. marina* **(B)**, *A. alba* **(C)**, and *A. officinalis* **(D)** under three different seasons. The arrows represent traits, while its length is based on the contribution of each trait to separate the accessions.

In A*. rumphiana*, the first two components (PC1 and PC2) explained 100% of the total variation (PC1: 78.61%, PC2: 21.39%). PC1 showed a positive correlation with secondary metabolites and compatible osmolytes and WSD, while carbohydrate, pigments, and RWC were negatively associated (**Figure 13A**).

In *A. marina*, the two significant main principal components account for all of the variance. Here PC1 accounted for 73.33% (**Figure 13B**). The secondary metabolites (TFC, TPPC) were positively associated with compatible osmolytes (proline, GB, mannitol, sorbitol) and environmental variables (average temperature, pH, salinity, TDS, and EC) but negatively associated with carbohydrate, pigments (Chl-a, Chl-b, and TC), and RWC. According to the Pearson correlation, TPC showed positive correlations with proline (*r* = 0.217), glycine betaine (*r* = 0.36), pH (*r* = 0.609), salinity (*r* = 0.443), TDS (*r* = 0.545), and EC (*r* = 0.69), whereas strong negative correlations were observed with carbohydrates (*r* = −0.525), chlorophyll-a (*r* = −0.869), chlorophyll-b (*r* = −0.995), and total chlorophyll (*r* = −0.94) (**Figure 9**).

In *A. alba*, PC1 and PC2 explained 100% of the variance, with 88.17% represented by PC1 (**Figure 13C**). The secondary metabolites (including TFC, TPPC), sugar alcohol (mannitol, sorbitol), GB, and proline were positively correlated with PC1. Sorbitol showed strong positive correlations with pH (*r* = 0.79), salinity (*r* = 0.89), TDS (*r* = 0.84), and EC (*r* = 0.723) but negative correlations with carbohydrates (*r* = −0.95), Chl-a (*r* = −0.78), Chl-b (*r* = −0.67), TC (*r* = −0.87), and RWC (*r* = −0.89) (**Figure 8**). In contrast, in *A. officinalis*, the first two components also explained 100% of the variation (PC1: 88.52%, PC2: 11.48%), whereas PC1 showed strong positive loadings for secondary metabolites (TPC, TFC, TPPC), compatible osmolytes (proline, GB, mannitol, sorbitol), and WSD, while carbohydrate, Chl-a, Chl-b, TC, and RWC were negatively associated. PC2 showed positive correlations with Man, Sor, Carb, Chl-b, TC, and WSD and negative correlations with Pro, GB, TPC, TFC, TPPC, and RWC (**Figure 13D**).

## 4 Discussion

Environmental factors (salinity, pH, tds, temperature, and EC) influenced oxidative stress, ionic balance, osmotic adjustment, and phenological behavior in mangrove plants, leading to adaptive responses such as reduced growth, lower chlorophyll content, and accumulation of compatible osmolytes. Therefore, *A. marina*, *A. alba*, *A. rumphiana*, and *A. officinalis* were selected to investigate the regulatory mechanisms associated with upwelling stress. We investigated the phenological patterns of mangrove species around the Digha Mohona mangrove vegetation and observed complex flowering and fruiting cycles (**Table 1**), with year-round flowering and fruiting activity during the rainy season, indicating that temporal variation regulates the reproductive strategies among mangrove species. Similarly, Songsom et al. (2019) reported that mangrove flowering begins from April to June, peaks during the monsoon, and continues into the late post-monsoon period. Mangrove phenology was regulated by cumulative rainfall at the Gulf of Thailand site. Some literature focused on mangrove phenology, revealing that *Bruguiera gymnorrhiza* flowered during the summer and rainy seasons, whereas *Rhizophora mucronata* flowered during summer and winter (Wang’ondu et al., 2013). However, the increased sea surface temperatures are known to decrease the leaf area in species like *Rhizophora mangle* (Agraz-Hernández et al., 2015), while shifts in precipitation patterns and air temperature significantly impact flowering and fruiting phenology across various mangrove species (Songsom et al., 2019). In previous studies, cumulative rainfall appears to be the primary environmental driver of mangrove phenology, and climate-change-related shifts in temperature, precipitation, and sea level may alter the flowering intensity and population dynamics (Van Der Stocken et al., 2022). In contrast, Kankong et al. (2021) noted that seasonal fluctuation regulated the leaf emergence rate and secondary cambial growth of *Avicennia alba*.

Salt glands are specialized organs in some halophytes that excrete the excess salt and maintain internal ionic balance. In this study, the leaf epidermis of *A. rumphiana* showed the highest number of SG and the lowest was in *A. alba* (**Figure 5**, **Table 2**). Although SG concertation did not vary seasonally, their numbers differed in a species-specific manner. Macnae (1968) reported that SG in *Avicennia* spp. form only under saline environments, whereas in *Aegiceras* spp. they appear irrespective of salt concentration. Joshi et al. (1975) further noted that *Avicennia* spp. typically grow in high-saline environments, while *Acanthus* spp. and *Aegiceras* spp. grow in low-saline regions.

Succulence, defined as the dilution of accumulated salts through increased leaf water content, represents a typical morphological adaptation to osmotic stress (Popp, 1995). In this investigation, the leaves of *A. rumphiana* and *A. officinalis* exhibited higher RWC during the monsoon season, likely due to succulence and salt accumulation (**Figure 7E**). These species also showed higher turgor weight to dry weight ratios, probably resulting from ion sequestration in vacuoles, unlike other salt-excreting species such as *A. alba* and *A. marina*, which maintain a lower ion level through SG activity. A previous research stated that large vacuoles maintain the leaf succulence and CO_2_ assimilation of *T. usneoides* (Kluge et al., 1973). Halophytes maintain osmotic balance under saline stress through physiological adaptations such as leaf succulence and active salt excretion (King et al., 1982; Breckle, 1990). However, trehalose accumulation regulates sucrose synthesis and allocation in wheat leaves under salinity stress conditions (Sadak, 2019).

Photosynthetic systems are sensitive to temperature and salinity variations; reduced chlorophyll content usually occurs under stress due to the inhibition of biosynthetic enzymes (Vosnjak et al., 2021). In our study, the pigment contents declined in *A. officinalis* and *A. rumphiana* during the pre-monsoon season, whereas *A. alba* and *A. marina* maintained a comparatively higher chlorophyll content. During the pre-monsoon season, increased salt accumulation in leaf tissues caused chlorophyll degradation (**Figures 7A–C**). During the monsoon season, the pigment contents significantly increased (*p* < 0.05). Plants are also able to construct a defense system that actively increases their chlorophyll contents and prevents decreases in photosynthesis and energy production (Zhang et al., 2019). n a previous work, *A. germinans*, *L. racemosa*, and *R. mangle* exhibited much greater variability in chlorophyll-a contents during the dry season than in the rainy season (Flores-De-Santiago et al., 2012; Flores-De-Santiago et al., 2016). Chlorophyll content and photosynthetic performance improved under low-salinity conditions (Zhang et al., 2019). Similarly, decline in leaf chlorophyll and anthocyanin level, respectively, was observed in *Alnus glutinosa* L. Gaertn and *Fraxinus angustifolia* Vahl under high-saline environments (Sariyildiz and Mohammed Ali, 2023).

In this study, the monsoon season reduced the salinity and enhanced the pigment production in *Avicennia* species. Total soluble sugar content (TSSC) varied seasonally, with the highest levels observed in *A. alba*, followed by *A. marina*, during the post-monsoon period. A previous work reported that the photosynthetic pigments utilize light energy to synthesize carbohydrates, which serve as essential substrates for respiration and metabolic processes in seaweeds (Khairy and El-Shafay, 2013). In contrast, soluble sugars maintained osmotic adjustment under salinity stress (Strogonov, 1964; Maas and Nieman, 1978; Doddema et al., 1986). An increase in the soluble sugar level in shoots often results from starch hydrolysis under water stress conditions (Jones and Qualset, 1984). Therefore, plants diverted carbon resources toward osmolyte synthesis to maintain osmotic balance under saline conditions, which may influence the plants’ growth.

Further investigation is required to clarify whether the formation of different osmolytes is linked to sodic or saline–sodic soil properties. Therefore, Mehta and Vyas (2023a) examined the precise composition and seasonal dynamics of osmolytes across a broader range of plant species under various salinity and sodicity conditions which could unveil more generalizable patterns and adaptive strategies. Different kinds of osmolyte accumulation were solely connected to flowering and seed development (Popp and Albert, 1995). In this result, there was greater osmolyte synthesis during the pre-monsoon season than the monsoon season. The composition and accumulation patterns differed among species as well as seasons. Similarly, Murakeozy et al. (2003) reported that species-specific responses reflect regulatory controls over osmolyte synthesis and accumulation, which are tailored to each plant genotype and its ecological niche. In contrast, compatible solutes maintain cellular osmotic balance while remaining metabolically non-disruptive (Galinski, 1993); they may also function as radical scavengers and osmoprotectants, stabilizing macromolecules under stress (Yeo, 1998).

In this study, proline accumulation was highest from late post-monsoon to late pre-monsoon in the leaves of all *Avicennia* species (**Table 6**). Increasing salinity in the pre-monsoon season promoted a higher proline accumulation in *A. rumphiana*, followed by *A. marina*, *A. alba*, and *A. officinalis* (**Figure 10A**). In previous studies, proline was accumulated through cytoplasmic solute adjustment that reduced the water potential, and osmotic gradient was maintained through water uptake under saline conditions, thereby contributing to salinity adaptation (Chaib and Benlaribi, 2006; Ibrahim, 2013; Hmidi et al., 2018). Conversely, proline can accumulate without disrupting the cellular metabolism and protects enzymes and cellular structures during stress (Jain et al., 2013; El Moukhtari et al., 2020). Proline accumulation may increase gradually during acclimation to salt stress until reaching a stable level (Parihar et al., 2014). The proline content increased in *A. marina* seedlings and saplings under salt stress (Cherian et al., 1999; Joseph et al., 2015).

GB and sugar alcohol also functioned as important compatible osmolytes. GB acts as an effective osmoprotectant, while polyols serve as free radical scavengers (Yeo, 1998; Mehta et al., 2023a, b; Adrian-Romero et al., 1998). In our study, the highest concentration of GB occurred from late post-monsoon to late pre-monsoon, with maximum levels in *A. rumphiana* and *A. officinalis* (**Figure 10B**). Similarly, the highest compatible osmolytes accumulate in other halophytes that are exposed to combined environmental stresses (Murakeozy et al., 2003). The results showed that sugar alcohol (mannitol and sorbitol) also increased with rising salinity from post-monsoon to pre-monsoon, with the highest accumulation in *A. rumphiana*, followed by *A. marina*, *A. alba*, and *A. officinalis*. Overall, osmolyte accumulation was strongly influenced by salinity intensity, seasonal variation, and species-specific adaptive capacity. These results emphasize the biosynthesis of osmolytes that helps to maintain cellular stability and protect the physiological function against osmotic stress (Watzka and Medina, 2018). The biosynthesis of various osmolytes is especially important for these halophytes, as it helps to enable survival in saline environments (Moslehi et al., 2025). By lowering the cellular water potential through the accumulation of osmolytes, mangroves are able to maintain active water uptake by the roots even at high soil salinity (Sudhir et al., 2022).

The pre-monsoon and post-monsoon periods showed elevated levels of secondary metabolites (phenols and flavonoids), indicating higher salinity stress during these seasons. Changes in the quantities of these metabolites most likely relate to the increased temperatures of these periods before the onset of the monsoon season, which affected the phenol quantities of all four plant species (**Table 6**). The plants tend to respond to salinity stress by adjusting the quantities of phenol and flavonoid derivatives, although secondary metabolite synthesis depends on species, tissue type, and NaCl concentration (Parihar et al., 2014). In literature, salinity stress leads to increased phenol and flavonoid derivatives to scavenge ROS and protect cells from oxidative damages (Bistgani et al., 2019). However, salinity stress can reduce the quantities of phenol and flavonoid derivatives due to disrupted metabolic activities and changes of resource allocations to overcome stress impacts (Rahimi and Biglarifard, 2011; Trivellini et al., 2014). In this study, the TPC changed depending on the species-specific manner and showed no significant change between the pre-monsoon and monsoon seasons. Similarly, total flavonoids were noted to be relatively stable between pre-monsoon and post-monsoon seasons (**Figure 5**).

## 5 Conclusions

The present study has revealed the prominent seasonal modulation of physiological and biochemical response of the four *Avicennia* species. All species exhibited effective salt tolerance mechanisms, supporting their saline coastal habitats and restoration. The accumulation of compatible osmolytes like proline, GB, mannitol, and sorbitol during the pre-monsoon season likely reflects higher salinity stress during this season. In contrast, chlorophyll and carbohydrate contents increased during the monsoon season, especially in *A. alba*, suggesting improved photosynthetic performance under comparatively favorable water conditions. The variation in SG occurrence in *A. rumphiana* epidermis, with seasonal changes in pigment and metabolite levels, regulated the species-specific physiological adaptive strategies to variable environmental conditions. Therefore, seasonal shifts in water availability and salinity modulate photosynthesis, osmotic adjustment, and metabolic activity. The data suggest that the seasonal variation was regulated relative to water content and metabolic activity, which helps in mangrove ecosystem resilience and restoration strategies in the face of climate change.

## Data Availability

The datasets presented in this study can be found in online repositories. The names of the repository/repositories and accession number(s) can be found in the article/supplementary material.
